# Switchable Broadband Terahertz Absorbers Based on Conducting Polymer‐Cellulose Aerogels

**DOI:** 10.1002/advs.202305898

**Published:** 2023-11-23

**Authors:** Chaoyang Kuang, Shangzhi Chen, Min Luo, Qilun Zhang, Xiao Sun, Shaobo Han, Qingqing Wang, Vallery Stanishev, Vanya Darakchieva, Reverant Crispin, Mats Fahlman, Dan Zhao, Qiye Wen, Magnus P. Jonsson

**Affiliations:** ^1^ Laboratory of Organic Electronics, Department of Science and Technology (ITN) Linköping University Norrköping SE‐601 74 Sweden; ^2^ School of Electronic Science and Engineering, State Key Laboratory of Electronic Thin Film and Integrated Devices University of Electronic Science and Technology of China Chengdu Sichuan 610 054 P. R. China; ^3^ Wallenberg Wood Science Center Linköping University Norrköping SE‐601 74 Sweden; ^4^ School of Textile Material and Engineering Wuyi University 22 Dongchengcun Jiangmen Guangdong 529 020 P. R. China; ^5^ Terahertz Materials Analysis Center (THeMAC) and Center for III‐N Technology, C3NiT‐Janzèn, Department of Physics, Chemistry and Biology (IFM) Linköping University Linköping SE‐581 83 Sweden; ^6^ Solid State Physics and NanoLund Lund University Lund SE‐221 00 Sweden; ^7^ Yangtze Delta Region Institute (Huzhou) University of Electronic Science and Technology of China Huzhou Zhejiang 313 001 P. R. China; ^8^ Stellenbosch Institute for Advanced Study (STIAS) Wallenberg Research Center at Stellenbosch University Stellenbosch 7600 South Africa

**Keywords:** conducting polymers, cellulose, terahertz, aerogels, redox tuning

## Abstract

Terahertz (THz) technologies provide opportunities ranging from calibration targets for satellites and telescopes to communication devices and biomedical imaging systems. A main component will be broadband THz absorbers with switchability. However, optically switchable materials in THz are scarce and their modulation is mostly available at narrow bandwidths. Realizing materials with large and broadband modulation in absorption or transmission forms a critical challenge. This study demonstrates that conducting polymer‐cellulose aerogels can provide modulation of broadband THz light with large modulation range from ≈ 13% to 91% absolute transmission, while maintaining specular reflection loss < −30 dB. The exceptional THz modulation is associated with the anomalous optical conductivity peak of conducting polymers, which enhances the absorption in its oxidized state. The study also demonstrates the possibility to reduce the surface hydrophilicity by simple chemical modifications, and shows that broadband absorption of the aerogels at optical frequencies enables de‐frosting by solar‐induced heating. These low‐cost, aqueous solution‐processable, sustainable, and bio‐friendly aerogels may find use in next‐generation intelligent THz devices.

## Graphical abstract

Aerogels made of conducting polymers and cellulose present excellent swtichable properties for THz optics. By switching the redox state of the areogels, the average broadband THz transmission can be signifciantlly modulated between 13% and 91%.

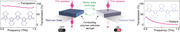

## 1 Introduction

The terahertz (THz) range from 0.1 to 10 THz represents an important and strategic band for both photonics and electronics research.^[^ ^1^, ^2^ ^]^ The myriad of opportunities for THz applications range from atmospheric remote sensing and astronomical explorations to security imaging and biomedical identification.^[^ ^3^, ^4^, ^5^, ^6^, ^7^ ^]^ A key component for these devices is THz absorbers, for example, for calibration targets enabling precise and accurate measurements.^[^ ^8^, ^9^ ^]^ THz absorbers have been put into practice for almost 40 years, with successes in various international collaborative projects, including applications in telescopes of Atacama Large Millimeter Array (ALMA),^[^ ^10^ ^]^ microwave instruments in Meteorological Operational Satellite–Second Generation (MetOp‐SG) and Ice Cloud Imager (ICI),^[^ ^8^ ^]^ and submillimeter wave instrument in Jupiter Icy Moons Explorer (JUICE) satellite.^[^ ^11^, ^12^ ^]^ However, the properties of most THz absorbers are static and possess no post‐fabrication tunability, excluding their applications from active and adaptive THz optics. Switchable materials that can modulate the absorption or transmission of THz signals are useful for intelligent calibration targets, dynamic attenuators for THz optical circuits, and effective modulators for electromagnetic interference, as demonstrated by multiple simulation results.^[^ ^13^, ^14^, ^15^ ^]^ Only a few materials have shown decent tunability in the THz range,^[^ ^13^, ^14^, ^16^, ^17^, ^18^, ^19^ ^]^ despite significant research progress in electromagnetic absorbers.^[^ ^20^, ^21^, ^22^, ^23^, ^24^, ^25^, ^26^ ^]^ Several of these systems focused on modulating THz properties via reflection rather than absorption, and often with limited tuning range.^[^ ^15^, ^18^ ^]^ For example, multiple layer devices based on graphene could tune THz reflection by 35% (between 50% and 85%).^[^ ^16^ ^]^ These devices also contained metal backplates and thereby did not provide modulation of THz transmission. Another study reported a graphene/quartz modulator with broadband tuning of reflection in the range from 0.5 to 1.6 THz, but the absolute modulation range of transmission remained < 20%.^[^ ^18^ ^]^ Other possible tunable materials like phase‐change materials (e.g., VO_2_) were studied mostly for tuning in the microwave range and the available tuning bandwidth is usually narrow.^[^ ^27^, ^28^ ^]^ For wearable or on‐skin applications, these materials or devices often also pose other issues associated with toxicity and limitations in terms of sustainability and bio‐friendliness.^[^ ^29^, ^30^ ^]^

Conducting polymers—as popularly explored in areas such as printed, flexible, and wearable electronics—form a promising class of materials to address these issues, thanks to their biocompatibility, sustainability, and, importantly, large tunability. Their tunable electrical and optical properties originate from the possibility to modulate their polaronic charge carrier density via their oxidation state. This feature has enabled applications such as electrochromic displays^[^ ^31^, ^32^, ^33^, ^34^ ^]^ and dynamic optical metasurfaces.^[^ ^35^, ^36^, ^37^, ^38^, ^39^ ^]^ Due to charge localization effects and molecular vibrations, conducting polymers further do not follow a Drude‐like optical conductivity (even in their most conducting state). Instead, the optical conductivity shows a broad peak centered in the mid infrared (IR).^[^ ^40^, ^41^, ^42^ ^]^ This means that their conductivity can be high in the THz and IR ranges despite having comparably low DC conductivity. Combined with their redox‐tunability, this offers unique unexplored opportunities for dynamically tunable THz materials. However, thin films of conducting polymers are limited to applications not requiring low THz reflection, because their high refractive index in the THz leads to considerable reflection.^[^ ^43^, ^44^ ^]^ We here introduce porosity to obtain a tunable THz material with low specular reflection in both states of the polymer. Conducting polymers were previously added to porous composite materials to enhance the absorption or shielding of radiation.^[^ ^45^, ^46^, ^47^ ^]^ However, only a few studies focused on their absorption or shielding properties in THz range^[^ ^43^, ^44^ ^]^ and they did not explore their redox switching capabilities for tuning THz radiation.

**Figure 1:**
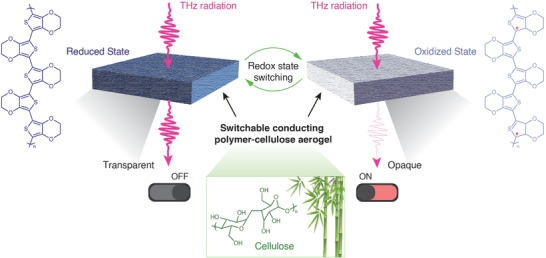
The THz transmission can be switched on and off by controlling the redox states of conducting polymer aerogels made from sustainable cellulose and PEDOT materials.

In this study, we introduce conducting polymer‐cellulose aerogels for low‐reflective broadband THz switchable absorbers (**Figure** **1**). Using a chemical vapor treatment, we could switch the pristine oxidized (conducting) aerogels to a reduced (insulating) state. For 1.5 mm thick aerogels with moderate conducting polymer content, this led to a drastic increase in broadband THz transmission from 13% to 91%, and with negligible specular reflection (<0.1%). In a more narrow spectral range around 1.2 THz, the transmission was modulated from only 2% to 90% upon switching. The process is reversible and the aerogels could be re‐oxidized to regain their THz transparency. To our knowledge, the presented large modulation range is superior to previously reported THz materials. We reason that the key to enabling these values is the tunable anomalous optical conductivity peak of the conducting polymer combined with reduced reflection and light‐trapping in the aerogel owing to its porous structure. With outdoor applications in mind, we further modified the surface hydrophilicity of the aerogels to avoid water uptake and we also demonstrated that solar‐induced heating can be used for efficient and passive removal of ice or frost layers. The aerogels are fabricated using aqueous solutions and do not require complex fabrication procedures, thereby holding great promise for large‐scale sustainable production at a low cost.

## 2 Results and Discussion

### 2.1 Aerogel Fabrication and Basic Properties

We prepared conducting polymer‐cellulose aerogels via freeze‐drying (Figure S1, Supporting Information) aqueous solutions of poly(3,4‐ethylenedioxythiophene):poly(styrenesulfonate) [PEDOT:PSS] and cellulose (see chemical structures in Figure S2, Supporting Information). Aerogels with different shapes could be fabricated by replacing the templating moulds or containers (Figure S3, Supporting Information). The electrical properties of these aerogels (in their pristine states) could be controlled by varying the ratio between PEDOT:PSS and cellulose (Table S1, Supporting Information), which also modified the microscale morphology of the aerogels (Figure S4, Supporting Information). Energy‐dispersive X‐ray (EDX) analysis shows that the PEDOT:PSS and cellulose were homogeneously distributed in the aerogel (Figure S5, Supporting Information). The PEDOT:PSS further formed an electrically conducting 3D network on the cellulose scaffold, which led to bulk electrical conductivities up to 0.24 mS cm^−1^ for materials with densities < 20 mg cm^−3^ (Table S1, Supporting Information). The percolation limit for electrical conductivity was very low, where aerogels with PEDOT:PSS weight ratio lower than 0.1% still exhibited a measurable electrical conductivity of ≈ 2.3 × 10^−3^ mS cm^−1^, sufficient for applications like antistatic charge coatings^[^ ^48^ ^]^ (Table S1, Supporting Information). Similar to aerogels based on other materials,^[^ ^49^ ^]^ the average diameter and size distribution of pores could be modulated by controlling the amount of water (Figure S6, Supporting Information) or freezing method (Figure S7, Supporting Information). The precise tuning of component ratios in precursor solution and processing conditions offers conducting polymer aerogels with tailorable microstructural, electrical, and optical properties to meet the requirements of versatile applications.

### 2.2 THz Properties of Pristine Conducting Polymer Aerogels

**Figure 2:**
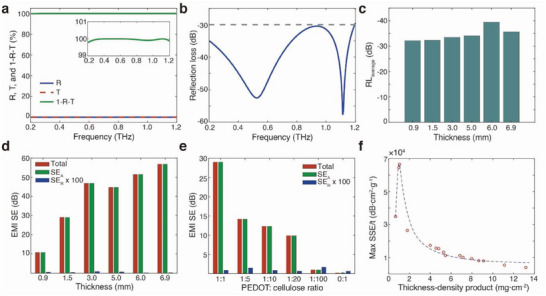
THz properties of the conducting polymer‐cellulose aerogels. a) *R*, *T*, and 1‐*R*‐*T* of an aerogel in the THz range (inset: enlarged *1‐R‐T* spectra in the THz range). b) THz specular reflection loss (RL) of the same aerogel. c) Comparison of average RL (RL_average_) for aerogels with different thicknesses. d) Comparison of EMI SE and its contributions from absorption SE_A_ and reflection SE_R_ (enlarged fo 100 times to be seen in the graph) for aerogels of different thicknesses. e) Comparison of EMI SE and its contributions from absorption SE_A_ and reflection SE_R_ for aerogels of different ratios of PEDOT:PSS to cellulose. f) The relation between max SSE/t and the product of thickness and density of the aerogels. (The dashed line is a fitted curve as a guide to the eye.) Conductive polymer‐cellulose aerogel presented in a and b has a thickness of 6 mm, and its component ratio of PEDOT:PSS to cellulose is 1:1.

Before studying switching capabilities, we first characterize the THz properties of different conducting polymer‐cellulose aerogels in their pristine oxidized state. **Figure** **2**a presents the THz transmission (*T*), specular reflection (*R*), and absorption (*A*) for a sample with a thickness of 6 mm. The absorption was here presented as *A*  =  1 − *R* − *T*, meaning that it also includes diffuse reflection and transmission. Complementary variable angle characterization indicates that such contributions are small, but may not be negligible (Figure S8, Supporting Information). Using *A*  =  1 − *R* − *T*, we calculate the average *A* to be 99.83% in the range between 0.2 and 1.2 THz, where in some ranges (e.g., 0.35 to 0.70 THz) the value was even > 99.99%. Such high values require extremely low specular reflection and indeed the specular reflection loss (RL) of the aerogels was lower than −30 dB in the entire spectral range, beyond the practical application requirement^[^ ^23^ ^]^ of −10 dB, as shown in Figure 2b. At 1.1 THz, RL reached a minimum of −58 dB, showing its high potential for blackbody absorbers that could be used for calibration targets in high‐precision measurements. The average reflection loss (RL_average_) for the entire range was −39.5 dB, representing one of the highest performance absorbers in the THz range.^[^ ^23^, ^24^, ^25^, ^50^, ^51^ ^]^ Decreasing the thickness of the aerogels caused only limited changes in RL_average_, and an aerogel with a thickness of only 0.9 mm still possessed RL_average_ < −30 dB (Figure S9, Supporting Information; Figure 2c). Even though thin aerogels showed slightly higher transmission and lower absorption compared to thicker gels, they still presented good electromagnetic interference (EMI) shielding efficiency (SE), as shown in Figure 2d. EMI SE was calculated through $\mathrm{EMI}\;\mathrm{SE}\;\left(\omega \right)=-10\times \log\left(E_{t}^{2}\left(\omega \right)/E_{ref}^{2}\left(\omega \right)\right)$z, where *E_t_* and *E_ref_* are the electric field strengths of the sample transmission and reference transmission (air) at the frequency ω. Our results show that aerogels with thicknesses > 0.9 mm are sufficient to guarantee EMI SE over 10 dB within a qualified bandwidth of 1.4 THz, sufficient for most THz applications (Figure S10, Supporting Information).^[^ ^22^, ^51^ ^]^

The excellent THz absorption is due to the presence of the PEDOT:PSS in the aerogel because pure cellulose aerogels without the conducting polymer only presented limited absorption < 15% (transmission > 85%), yet still with low RL (Figure S11, Supporting Information). In turn, this enabled gradual control of the THz absorptivity by varying the ratio between PEDOT:PSS and cellulose, as shown in Figure S12 (Supporting Information). For an aerogel thickness of 1.5 mm, a ratio of 1:20 ensured an EMI SE of 10 dB (sufficient for practical use, Figure 2e), significantly reducing the required amount of PEDOT:PSS (1 mg for a 4 cm^3^ aerogel) and thus the overall cost of the aerogels. The ratio had to be varied significantly to induce large changes in the THz properties, meaning that the performance had a strong tolerance to small variations in the material ratio. For example, a difference from 1:5 to 1:10 caused only limited changes in the THz response (Figure S12, Supporting Information). This may partly be associated with the low percolation limit of PEDOT:PSS for electrical conductivity, as discussed above. By varying the water ratio, we could vary the pore structure and investigate its influence on the THz absorption (Figure S13, Supporting Information). THz radiation at low frequencies (or long wavelengths) could partly be reflected in aerogels with smaller pore sizes (i.e., water ratio of 168), while a large fraction was transmitted through aerogels with larger pore sizes (i.e., water ratio of 982). Aerogels made by liquid nitrogen cooling instead of freezer cooling showed similar performance, despite the difference in microstructure (Figure S7, Supporting Information). A common performance indicator for practical applications is the specific SE per unit volume (SEE/t), which is EMI SE normalized by the product of density and thickness. As shown in Figure 2f, we find that in a certain range, the SSE/t of our aerogels could be significantly increased by decreasing the aerogel thickness or density (with a higher water ratio). The maximal SSE/t achieved with our aerogels was 6.6  ×  10^4^ dB·cm^2^·g^−1^ (with thickness‐density product of 1.05 mg·cm^−2^), which is among the highest SSE/t reported in the literature.^[^ ^24^, ^50^, ^51^, ^52^ ^]^ Further decreasing the thickness‐density product reduced the maximal SSE/t, owing to challenges in maintaining aerogel properties and thickness homogeneity for ultrathin and low‐density aerogels.

We compare our aerogels with other (non‐tunable) high‐performance THz absorbers reported in literature.^[^ ^28^, ^29^, ^30^ ^]^ The comparison is made based on six key attributes, including qualified bandwidth, SSE/t, RL_average_, sustainability, ease of fabrication, and cost effectiveness, as exhibited in Table S2 (Supporting Information). Despite a lower maximal SSE/t compared to Graphene/poly(methyl methacrylate) [or Graphene/PMMA] laminates^[^ ^51^ ^]^ (3 × 10^5^ dB·cm^2^·g^−1^), our conducting polymer aerogels show the lowest RL_average_, which is desired for applications requiring low surface reflection. The fabrication of our aerogels requires no fluoric acid treatment^[^ ^50^ ^]^ no high‐temperature treatment^[^ ^52^ ^]^ and no multiple repeating steps.^[^ ^51^ ^]^ Furthermore, PEDOT:PSS and cellulose, as the main raw materials, are sustainable, bio‐friendly, and abundant materials at low prices. The fact that both materials have already been broadly utilized in flexible, wearable, and bio‐electronic devices^[^ ^53^, ^54^, ^55^, ^56^ ^]^ lowers the barrier to integrating our THz materials into such systems. We further note that the fabrication method of our conducting polymer‐cellulose aerogels is robust and suitable for large‐scale manufacturing, posing no strict requirements on the precise control of material ratio, water amount, and thickness.

We also studied the optical properties in the microwave band (25–40 and 75–110 GHz), as presented in Figure S14 (Supporting Information). Compared to the THz range, the overall absorption (1‐*R*‐*T*) in the microwave range was weaker, yet remained close to 90% down to 25 GHz. The RL for 75–100 GHz was < −30 dB, while a RL_average_ of −14.6 dB was found in the lower frequency part < 40 GHz. Aerogels made of pure PEDOT:PSS showed similar performance as aerogels blended with cellulose and GOPS. By contrast, aerogels made of pure cellulose showed significantly lower microwave absorption, with transmission remaining > 20% in the whole measured range. The results highlight that the PEDOT:PSS and its conductive properties are essential also for microwave absorption. Other potential factors, such as the macrostructures, may also become dominant in this range, as revealed by other studies.^[^ ^57^, ^58^ ^]^

### 2.3 Redox‐Switchable THz Properties

**Figure 3:**
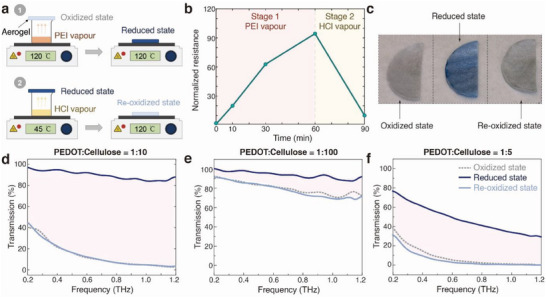
Redox state tunability and switchable THz transmission of conducting polymer‐cellulose aerogels. a) PEI (1) and HCl (2) vapor treatment for conducting polymer‐cellulose aerogels. b) Resistance of conducting polymer‐cellulose aerogel at different redox states (normalized based on its initial resistance at oxidized state). c Images of the aerogels at different redox states. The aerogel used for panel (a–c) had a thickness of 1.5 mm and PEDOT:PSS to cellulose ratio of 1:10. Panels d–f) present reversible tuneable THz transmission for aerogels, for three different ratios of PEDOT:PSS to cellulose of (d) 1:10, (e) 1:100, and (f) 1:5. The THz transmission at three different states (oxidized, reduced, and re‐oxidized) are displayed. The tuning ranges are highlighted by a light pink shading.

The electrical conductivity provided by PEDOT:PSS networks forms the main absorption mechanism of the aerogels, which is known as the electrical conduction loss.^[^ ^59^ ^]^ For PEDOT:PSS, the electrical properties originate from polaronic charge carriers, with electrical neutrality balanced by counterions.^[^ ^60^ ^]^ Modulating the carrier concentration via the doping level (or redox state) can significantly alter the electrical and optical properties of conducting polymers, even between metallic and dielectric states.^[^ ^31^, ^32^, ^35^, ^36^ ^]^ Here, we adopt a chemical approach to demonstrate theredox state switchability of the conducting polymer aerogels and to investigate their switchable optical behavior in the THz range. Similar chemical approaches have been broadly used for various optically switchable systems, including metasurfaces based on magnesium^[^ ^61^ ^]^ and titanium oxides,^[^ ^62^ ^]^ and conducting polymer nanoantennas.^[^ ^35^ ^]^ We initially focus on aerogels with PEDOT:cellulose ratio of 1:10. **Figure** **3**a schematically depicts the reversible switching concept, where the aerogels are exposed to the vapor of certain chemical agents in sealed containers. For reduction, we used the vapor of branched poly(ethylenimine) (PEI) heated at 120°C (Figure S15, Supporting Information). Exposure to PEI vapor for ≈ 10 min led to an increase in electrical resistance of > 20 times, and a change of > 2 orders of magnitude could be achieved with 60 min treatment (Figure 3b). This huge change in DC conductivity was accompanied by a color transition of the aerogel from light grey to blue (Figure 3c). The reduction is associated with the disappearance of polaronic charge carriers and the emergence of neutral state interband transition in PEDOT:PSS, which has been widely reported for PEDOT‐based thin films.^[^ ^35^, ^63^ ^]^ Importantly, the switching process is reversible and the aerogel could be re‐oxidized and retrieve its electrical conductivity by a 30 min hydrochloride (HCl) vapor treatment (Figure 3b). Similarly, the color of the aerogel returned to its initially light grey appearance upon re‐oxidation. As detailed in Note SI (Supporting Information), X‐ray photoelectron spectroscopy (XPS) confirmed that the switching involves varying the PEDOT in the aerogel between its charged and neutral states, and also provided more detailed information about the switching mechanism (Figure S16a–c, Supporting Information).

We will now demonstrate that the aerogels can act as switchable THz materials with very large modulation range. Figure 3d presents the measured THz transmission of a 1.5 mm thick aerogel before and after PEI vapor treatment (PEDOT to cellulose ratio 1:10). The average THz transmission increased all the way from 13% in the pristine oxidized state to 91% in the reduced state. An even more drastic modulation was observed in the higher frequency range, from around only 2% to 90% transmission at 1.2 THz. To our knowledge, these values outperform previously reported tunable broadband THz materials. We further note that the transparent state shows transmission similar even to that of pure cellulose aerogels (Figure S11, Supporting Information). Both states showed low specular reflection in the entire range (Figure S16, Supporting Information), indicating that the changes in transmission are primarily due to variations in absorption. This further confirms that the conductivity of the polymer plays the dominant role in the THz absorption of the aerogels. Re‐oxidizing the aerogel led to THz transmission that almost overlapped with that of the initial oxidized material (before switching). The results confirm that the broadband THz transmission of the aerogels can be reversibly controlled, with a large tuning range of almost 80 percentage points (≈ 10% to 90%, Figure 3d). The redox tuning could be repeated over multiple cycles, as demonstrated by electrical resistance characterizations (here measured for a total of 5 cycles, Figure S17, Supporting Information). We also note that the aerogels treated by a cycle of PEI and HCl vapor treatments remained stable in the air, with no observed degradation in electrical conductivity for > 20 days. The sensitivity to certain vapors also indicates the possibility of using these aerogels for gas sensing applications. Compared to other types of switchable THz devices,^[^ ^16^, ^18^, ^19^ ^]^ our chemical modulation approach is rather slow and may be inconvenient for some applications. Future research may therefore focus on implementing other tuning modalities, and it is promising that conducting polymer systems have shown compatibility with electrical tuning at video rates.^[^ ^64^, ^65^ ^]^ We further note that not all applications require fast switching, so the current materials may find use in practical applications, for example, for low‐speed tunable attenuators to modulate the light intensity of THz sources. In addition, our aerogels provide a good optical memory effect, where the state of the aerogels is non‐volatile, which is also an attractive property for many applications (Figures S18 and S19, Supporting Information).

We further investigated the switching capability and THz tuning range of aerogels with different component ratios (but the same thickness of 1.5 mm). The results are presented in Figure 3e,f and Figures S20 and S21 (Supporting Information). For samples with low PEDOT:PSS to cellulose ratio (1:100), the electrical resistance initially increased upon PEI treatment, but then decreased for longer exposure times (Figure S21, Supporting Information). This could partly be an artifact from re‐oxidation in the air after treatment, which may have a larger effect on aerogels with low PEDOT concentrations. Yet, the aerogel with a 1:100 ratio still provided clear reversible switching capability in terms of broadband THz transmission, with average THz transmission switching between 78% and 94% (Figure 3f). As expected, the transmission was higher in both redox states for this aerogel compared to that of the standard aerogels with higher PEDOT content (ratio of 1:10). Aerogels with yet higher PEDOT content (1:5) could also be switched but showed lower THz transmission in both states, with modulation range of 42 percentage points, between 5% and 47% (Figure 3e). Hence, we found a ratio of 1:10 to provide the largest modulation range for these samples, by containing sufficient content of PEDOT to ensure high absorption in the oxidized state, while still being able to become highly transmissive in the reduced state. Notably, the tuning range (and optimized ratio) will also depend on aerogel thickness. In general, aerogels with larger thicknesses but the same material ratios could achieve similar results but narrower tuning ranges. For shorter‐wavelength light in the infrared and visible, the aerogels remained highly non‐transmissive in both states (Figure S22, Supporting Information). Utilizing such low transmission in both states at higher frequencies makes the aerogels suitable not only as tunable THz absorbers but also as switchable high pass filters.

To our knowledge, the presented aerogels outperform materials previously shown to possess switchable properties in the THz range (Table S2, Supporting Information).^[^ ^16^ ^]^ Reports of graphene‐based cavity devices presented tunable THz reflection between ≈ 50% and 85%, which would translate to a variation in a combined THz absorption and transmission not exceeding the range between 15% and 50%.^[^ ^16^ ^]^ Hence, the tuning range was smaller than for our aerogels, although the graphene‐based devices had the advantage of being tuned electrochemically.^[^ ^18^, ^19^ ^]^ Similarly, studies on aerogels composed of phase change materials exhibited switching behavior, but with a more limited tuning range.^[^ ^13^, ^14^ ^]^ For example, Chang et al. reported RL tuned from −17 to −54 dB, corresponding to a modulation in absorption in the range from 98% to ≈100%.^[^ ^28^ ^]^ In addition, the tuning often covered a relatively narrow band with frequencies mostly located in the microwave range instead of in the THz range.^[^ ^27^, ^28^ ^]^ Conducting polymer‐cellulose aerogels thereby shows great potential as switchable THz materials, including not but limited to absorbers and switchable broadband attenuators or filters.

### 2.4 Understanding the THz Switching Behavior

**Figure 4:**
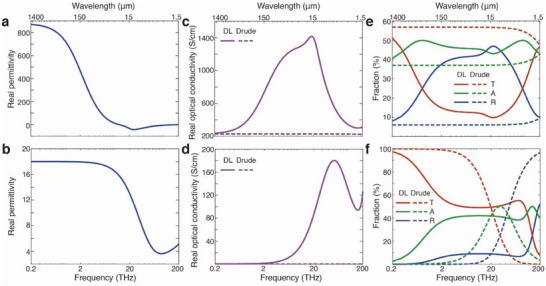
Optical parameters and calculated absorption for PEDOT:PSS films. a,b) Real permittivity dispersion of PEDOT:PSS‐GOPS films with (a) and without PEI vapor treatment (b). c,d) Real optical conductivity dispersion of PEDOT:PSS‐GOPS films with (c) and without PEI vapor treatment (d). e,f) Calculated absorption, reflection, and transmission curves for PEDOT:PSS‐GOPS films with (e) and without PEI vapor treatment (f). Solid lines are results from Drude–Lorentz model, while dashed lines are results from Drude model. For calculation in e to f, the film thicknesses were 76 nm.

To reveal the underlying reasons for the strong switchable THz response of the aerogels, we investigated the optical parameters of PEDOT:PSS thin films with and without PEI treatment (Figure S23, Supporting Information).^[^ ^40^ ^]^ The permittivity and optical conductivity of pristine PEDOT:PSS and PEI‐treated PEDOT:PSS were derived using ultra‐wide spectral range ellipsometry and a Drude–Lorentz model (**Figure** **4**a,d; Figure S24, Supporting Information). In agreement with previous studies on conducting polymers,^[^ ^41^, ^42^, ^66^ ^]^ the results show a broad optical conductivity peak centered in the mid infrared for the oxidized film (Figure 4c). This peak can be attributed to charge localization effects and molecular vibrations.^[^ ^40^, ^41^, ^42^, ^43^, ^60^, ^67^ ^]^ As shown in Figure S24e,f (Supporting Information), this broad peak was also visible in the imaginary permittivity in the mid infrared with tails extending into the THz regime. The real part of the optical conductivity is associated with absorption^[,60]^ and the tail of the peak in the THz can thus increase the effective absorption also in this spectral range, even for modest DC conductivities. The optical conductivity peak decreased dramatically upon reduction, which agrees with a lower THz absorption for the reduced materials (Figure 4d). For a freestanding film in the air with thickness much smaller than the wavelength of the electromagnetic radiation, the transmission (*T*), absorption (*A*), and reflection (*R*) at normal incidence can be estimated using the following simplified formulae^[^ ^68^ ^]^:

(1) $$T=4g^{2}/{\left(1+2g\right)}^{2}$$

(2) $$A=4g/{\left(1+2g\right)}^{2}$$

(3) $$R=1/{\left(1+2g\right)}^{2}$$

where the frequency‐dependent coefficient *g* is expressed by^[^ ^68^ ^]^:

(4) $$g=1/\left(\sigma \cdot t\cdot Z_{0}\right)$$

where *σ* is the real optical conductivity, *t* is the film thickness, and *Z_0_* is the impedance of free space (377 Ω). Figure 4e,f presents the obtained transmission, absorption, and reflection curves for a PEDOT:PSS film with and without PEI treatment. In the oxidized state of the film (denoted as “DL” [for Drude–Lorentz], see Figure 4e), the overall absorption is > 40%, with a maximum of ≈ 1 THz. The reflection varies from ≈10% to ≈25% in the THz range. For comparison and to illustrate the contribution of the conductivity peak to these results, we also calculated the transmission, absorption, and reflection of artificial material with optical conductivity only based on the Drude component (denoted as “Drude”). Hence, this “Drude material” has the same DC conductivity as derived for the conducting polymer using the DL model, but it does not possess the optical conductivity peak and thereby more resembles a traditional metal than a conducting polymer. For this Drude material, both absorption and reflection are lower compared to calculations for the real DL material. This corroborates that the anomalous optical conductivity peak of the conducting polymer provides an important contribution to the THz absorption. As a result, and in contrast to conventional metals, this relaxes the requirement of having high DC conductivity to obtain high THz absorption for conducting polymer materials, including for our aerogels. Mechanistically, the behavior can be understood from the decreasing carrier diffusion length toward higher frequencies,^[^ ^60^, ^69^, ^70^ ^]^ resulting in THz radiation mostly probing charge transport at short lengths scales locally within small regions of the material (e.g., within PEDOT:PSS aggregates). In turn, high optical conductivity can likely be obtained even without the need for reaching macroscopic percolation within the material, as critical for DC conductivity.^[^ ^71^ ^]^ In the reduced state (Figure 4f), the reflection in the THz range is low (<10%) and the absorption is mostly < 20%. Hence, already a flat thin PEDOT:PSS film should be able to provide modulation of THz transmission via changes in reflection and absorption. For the aerogels, surface reflection from the material is suppressed due to the porous structure, improving the impedance matching with air. In addition, the multiple scattering sites inside the aerogels effectively trap light in the material until absorbed by the oxidized material. Reducing the PEDOT in the aerogels leads to lower reflection as well as lower absorption at each interface in the porous medium, which agrees with the increased transmission through the aerogel after PEI treatment.

Figure S25 (Supporting Information) presents calculations for additional artificial materials based on the Drude–Lorentz model with various optical conductivity dispersions (by changing the charge carrier mobility). As for metallic conductors, there is an optimal value for maximized absorption where the optical conductivity‐thickness product should be ≈ 5.31 mS (2/*Z*_0_ from Equation (2)). Based on the Drude model, the optical conductivity at low frequency range of a metal is almost identical to the DC conductivity. Therefore, the DC conductance needs to be ≈ 5.31 mS for a Drude metal film to have high THz absorption (Figure S25f, Supporting Information). However, the anomalous peak of the conducting polymer widens the optimal range in DC conductivity. Indeed, as shown in Figure S25f (Supporting Information), the film THz absorption of materials with DC conductivity of 80 S cm^−1^ can be improved from 18% (Drude) to 47% (Drude–Lorentz or DL) with the contribution from the optical conductivity peak. Materials with higher DC conductivity of 1600 or 8000 S cm^−1^ showed a slight decrease in the average THz absorption if adding the Lorentz oscillators.

### 2.5 Environmental Tolerance: Hydrophilicity Modulation and Solar Heating Properties

Both PEDOT:PSS and cellulose are known to be hydrophilic, which is important in enabling aqueous solution processing for printed electronics.^[^ ^60^, ^69^ ^]^ Some studies also demonstrated that both materials can be hygroscopic in air,^[^ ^72^ ^]^ and the incorporation of water into the aerogel structure could cause collapse and reduce the electrical conductivity of PEDOT films. In addition, the switchable THz absorbers will in some practical applications be placed outdoors and required to withstand different environmental conditions, including but not limited to humid and cold climates. Thus, implementing the capability of the aerogels to maintain stable functions in different environments or to recover their high‐performance properties would be highly useful. We here demonstrate that through certain treatments, our conducting polymer aerogels can sustain extreme conditions.

**Figure 5:**
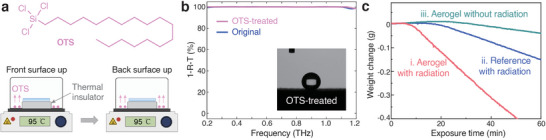
Hydrophilicity modification and de‐icing properties of aerogels. a) Chemical structure of OTS and the process flow of OTS treatment. b) THz absorption curves for aerogels with and without OTS treatment (inset: contact angle measurement of OTS‐treated surface of the aerogel. c) Weight reduction curves of aerogel and reference samples with ice pieces on top under different situations.

To decrease the hydrophilicity of the switchable THz absorbers, we applied octadecyl trichlorosilane (OTS) vapor treatment to a conducting polymer aerogel (6 mm thickness and PEDOT to cellulose ratio of 1:1). OTS is a widely used small molecule (structure in the inset of **Figure** **5**a) for hydrophobic surface modification.^[^ ^73^ ^]^ In brief, trichlorosilane groups in OTS can react with the ─OH groups (rich in PSS and cellulose) to form chemical bonds^[^ ^74^ ^]^ and be attached to the porous surfaces creating a self‐assembled monolayer. The alkyl chains of OTS therefore can make the whole surface hydrophobic, as demonstrated by first principle calculations.^[^ ^75^, ^76^, ^77^ ^]^ Indeed, 30 min vapor treatment could create a highly hydrophobic surface, with a water contact angle of 151° (see Figure 5b). The hydrophobic layers remained stable for at least 4 weeks without any sign of reduction of the hydrophobicity (Figure S26, Supporting Information). We simulated rainy outdoor conditions by pouring water onto the aerogel or placing the entire aerogel into a water bath (Video S1, Supporting Information). We found that water could hardly penetrate into the aerogel, despite its high porosity. A small mechanical vibration could easily throw away water droplets on the aerogel surfaces and no water trace was left on the surfaces. Examining the THz spectra of OTS vapor‐treated aerogels showed that their absorption (1‐R‐T), specular reflection (R), and transmission (T) remained almost identical to non‐treated aerogels (Figure 5b; Figure S27, Supporting Information). OTS vapor treatment can therefore be suitable for the use of the tunable THz conducting polymer‐cellulose aerogels for applications in humid or rainy environments.

Coldness forms another key challenge for outdoor THz applications since THz detection can be significantly affected by formation of frost or ice layers on the device's surface.^[^ ^78^ ^]^ Avoiding the formation of frost layer or eliminating as‐formed ice layer would be desired for practical use. Our aerogels exhibit high absorption in the solar spectrum thanks to the polaronic charge carriers of the PEDOT,^[^ ^79^ ^]^ as previous studies utilized for solar‐powered photothermal conversion.^[^ ^80^ ^]^ Thermal camera imaging indicates that the aerogels possess a low thermal conductivity in their vertical direction (normal to the sample surface), because light‐induced temperature increase was mostly localized to the top surface (Figure S28, Supporting Information). The combination of good photothermal conversion and heat localization effect enables the de‐icing (de‐frosting) properties of the conducting polymer aerogels, which we will discuss below.

As shown in Figure 5c and Figure S29a (Supporting Information), we placed a piece of ice (with a weight of ≈ 1.0 g and area of 3.3 cm^2^) on top of the aerogel under simulated solar illumination (i), and measured the weight reduction of the whole system. For comparison, we introduced two reference samples, in the form of a petri dish under illumination (ii) and an aerogel without illumination (iii), with ice pieces of similar weight and size placed on top. In the initial stage, the overall weight showed a slight increase, likely due to condensation of water vapor from the air (Figure 5c). After ≈ 6 min, sample (i) exhibited a sharp decrease in weight (Figure S29a, Supporting Information), while the decrease started after significantly longer times for samples (ii, 17 min) and (iii, 33 min). As a result, the illuminated aerogel sample (i) had already melted and evaporated some of the ice after 10 min, while the weights of the other samples were still increasing. The right part of Figure S29a (Supporting Information) presents photographs of the samples after 20 min, showing that the ice pieces had melted into large water droplets on samples (i) and (ii), while sample (iii) still had a large ice piece on top. While forming such large ice pieces may be less likely in real outdoor conditions, a more likely scenario would be that a thin layer of frost is formed on top of or around the aerogel. To simulate the frosting process, we placed aerogels on top of a beaker filled with liquid nitrogen, and it took ≈ 5 min to create a frost layer (Figure S29b, Supporting Information). The aerogels were then exposed to solar illumination and it took only ≈ 60 s for the aerogels to remove the frost layer around them. It took another 120 s to melt the area around them, while the frost on aerogel areas outside the illumination spot remained unchanged during the measurement time (Figure S29c, Supporting Information). Our results demonstrate promising de‐icing (and de‐frosting) properties of conducting polymer‐cellulose aerogels, due to their strong solar absorption, low thermal conduction, and heat localization properties, holding promise for practical application in complex environments with high humidity and low temperature.

## 3 Conclusion

Our study demonstrates the use of conducting polymer‐cellulose aerogels for high‐performance switchable broadband THz absorbers. The strong THz absorption in its pristine (oxidized) state is due to the polaronic charge carriers of the conducting polymer and the anomalous optical conductivity peak. Low influence from reflection is further possible thanks to the porous nature of the material. Controlling the redox states of the polymer enabled exceptionally large and reversible modulation of the THz transmission, in the range from 2% to 90% transmission at 1.2 THz and from 13% to 91% as average values in a broader range. To our knowledge, this is the largest achieved modulation capability for broadband THz transmission materials reported to date. Future developments will benefit from the ability to also switch conducting polymer systems by electrical potentials,^[^ ^36^, ^81^ ^]^ even at videorates,^[^ ^39^, ^64^, ^65^ ^]^ which further strengthens the relevance of these materials for practical applications. In addition, we demonstrate modification of surface hydrophilicity and de‐icing and de‐frosting properties of the aerogels, further improving their suitability for practical applications. Potential applications include tunable attenuators (neutral density filters) for THz light sources or dynamic protecting shields for wireless electronic devices which can selectively prevent unwanted electromagnetic interference based on need. The promising switchable THz properties of conducting polymer‐cellulose aerogels will offer various possibilities for further interdisciplinary studies, for example, combining with their existing use in wearable bioelectronics, paving the way toward on‐body THz communication systems.

## 4 Experimental Section

### Conducting Polymer Aerogel Fabrication

Conducting polymer PEDOT:PSS (PH1000, Heraeus Clevios, 1.3 wt%), nanofibrous cellulose (carboxymethylated, RISE Innventia AB, 1.0 wt %), and GOPS (Sigma–Aldrich, 98 wt%) were mixed in a solid ratio of 1:1:0.2 for standard samples. ULTRA‐TURRAX T‐10 dispenser was used for solution mixing and the obtained suspension was poured into a certain container or mould, which was later transferred to a freezer at −20°C for 5 h. The frozen solution was then freeze dried under −50°C at 200 µbar for ≈ 24 h (Benchtop Pro, SP Scientific freeze dryer). The obtained aerogels were then thermally annealed in an oven at 140°C for 30 min to crosslink GOPS with PEDOT:PSS and cellulose. DMSO vapor treatment was applied to increase the electrical conductivity of the aerogels as used in the previous studies. The redox state tuning of the conducting polymer aerogels was realized by oxidizing/reducing vapor treatment. For the reduction (de‐doping) process, the vapor of branched poly(ethylenimine) (PEI, Sigma–Aldrich, M_w_ ≈ 800) was used by heating a vial containing PEI at 120°C for 10 to 60 min, followed by a thermal annealing step at 120°C for 10 min. For the oxidation (re‐doping) process, the vapor of hydrochloric acid (HCl, Sigma–Aldrich, 37%) heated at 45°C was used for 30 min, followed by thermal annealing at 120°C for 10 min. For surface hydrophilicity modification, OTS vapor treatment was used.^[^ ^73^ ^]^ Briefly, the aerogels were placed in a sealed glass petri dish with a few drops of OTS added around them. The petri dish was then placed on a hotplate at a temperature of 95°C for 1 h. After that, the aerogels were flipped over and another 1 h treatment was applied to ensure that both surfaces of the aerogels were treated by OTS vapor.

### De‐Ice and Defrost Experiments

For de‐ice experiment, ice pieces with similar weight were placed on top of the aerogel and reference. The time‐dependent weight variations of samples were monitored by a digital balance (Ohaus) under 1‐sun illumination with a solar simulator (Newport). The temperature distribution of the aerogels was also characterized by an infrared camera (FLIR Thermal Camera ThermoVision A320G), and the emissivity of the aerogel was assumed to be 1. For defrost experiment, a beaker containing liquid nitrogen was covered by a piece of black aluminum foil (Rosco Matte Black Cinefoil) leaving small gaps as outlets for nitrogen gas. The aerogels were placed on top of the foils and a thick frost layer rapidly formed within a few minutes. The setup was then moved to the solar simulator for 1‐sun illumination and the whole defrost process was recorded by a Huawei Mate 20 Pro phone.

### Electrical Conductivity Characterization

The thicknesses of the aerogels were measured by a Vernier calliper. For electrical wiring, it was difficult to directly connect the aerogels with needle electrodes (due to the limited contact area) or conductive Cu tapes (due to its relatively strong adhesion that can damage the surfaces of aerogels). Therefore, custom‐made cavity structures were used to measure the resistance, as schematically presented in Figure S30 (Supporting Information). Briefly, the aerogel for measurement was placed in a cavity sandwiched by two metal electrodes (Au thin films with thicknesses of 120 nm). The height of the cavity can be tuned by replacing the plastic spacer with different thicknesses (e.g., 2, 5, or 8 mm) that were smaller than the height of the aerogel. The electrical resistance of the aerogel can be obtained by interpolation of the measured values at different thicknesses to its original thickness. The aerogels in this study were elastic and the thickness of the aerogel can completely recover after each measurement (no plastic deformation occurs). The obtained results using this method are highly reproducible. In the previous studies, it was demonstrated that electrical resistances of the aerogels had a linear dependence on the external pressures (or thicknesses) within the elastic range.^[^ ^82^, ^83^ ^]^ Thus, the linear interpolation approach used here can give reliable results for the electrical resistance of the aerogels. The electrical conductivity is calculated by σ  = *t*/(*R* · *A*), where *t* is the thickness, *R* is the electrical resistance, and *A* is the surface area of the aerogel.

### Structural and Chemical Characterization

Morphology characterization was performed by a scanning electron microscope (Zeiss Sigma 500). Contact angle characterization was carried out by a contact angle goniometer (Ossila). X‐ray photoemission characterization was carried out by using a Scienta ESCA 200 spectrometer under ultrahigh vacuum conditions at a base pressure of 1 × 10^−10^ mbar. The measurements were done with a monochromatic Al Kα X‐ray source, which provides photons with an energy of 1486.6 eV. The spectra were normalized to the C1s peak. Photos and videos of the aerogels in the study were taken by a Huawei Mate 20 Pro phone.

### THz Characterization

The Terahertz time‐domain spectroscopy (THz‐TDS) was measured by a commercial all‐fiber based system (Fico, Zomega Co., Ltd.). The measurement was performed at room temperature. The humidity in the characterization room was controlled in a low range (< 5%). The effective spectrum range is 0.2–1.6 THz, and the repetition frequency is 1 kHz. The testing time step was ≈ 0.067 ps throughout the whole experiment. The THz absorption and reflection measurement was carried out using the above equipment and the details of the setup can be found in the previous reports.^[^ ^24^, ^25^ ^]^ The phase and amplitude of the frequency‐domain spectra of the aerogels were extracted through fast Fourier transform algorithm. The THz transmission and reflection were calculated using $T\;\left(\omega \right)=E_{t}^{2}\;\left(\omega \right)/E_{ref1}^{2}\left(\omega \right)$ and $R\;\left(\omega \right)=E_{r}^{2}\;\left(\omega \right)/E_{ref2}^{2}\left(\omega \right)$, where *ω* is the angular frequency, and *E_t_*, *E_r_*, *E_ref1_*, and *E_ref2_* were the electric field strengths of sample transmission, reflection, and corresponding reference transmission (air) and reflection (Al plate). Reflection loss (RL) of the aerogels was calculated using $RL=10\times log(E_{r}^{2}\left(\omega \right)/E_{ref2}^{2}\left(\omega \right))$. For electromagnetic interference (EMI) shielding efficiency (SE), the formula is $EMI\;SE\;\left(\omega \right)=-10\times log\left(E_{t}^{2}\left(\omega \right)/E_{ref1}^{2}\left(\omega \right)\right)$. The corresponding EMI SE due to reflection and absorption was obtained by $SE_{R}\;\left(\omega \right)=-10\times log\left(1-E_{r}^{2}\left(\omega \right)/E_{ref2}^{2}\left(\omega \right)\right)$ and *SE_A_* (ω) =  *EMI* *SE*(ω) − *SE_R_* (ω). EMI SE values were averaged on the measured frequency range for comparison. The average reflection loss (RL_average_) is defined as the ratio between the integrated RL value over the measured frequency range and the measured frequency bandwidth.

### Millimeter Wave Characterization

For the measurement in the ranges 25–40 GHz and 75–110 GHz, a vector network analyser (Keysight N52278 and FEV‐1‐TR‐0006 frequency extender module) was used. All samples were placed on a 5 mm thick standard Al plate in the test and all the measurements were conducted at room temperature.

### Spectroscopic Ellipsometry Measurement

PEDOT:PSS thin film samples were measured at room temperature under normal ambient conditions. The details of measurement are available in our previous studies.^[^ ^35^, ^40^ ^]^ Briefly, a RC2 spectroscopic ellipsometer (J. A. Woollam Co.) and an IR‐VASE spectroscopic ellipsometer (J. A. Woollam Co.) were used for UV–vis–NIR range (210–1690 nm) and middle IR range (7813–230 cm^−1^). For THz range, the THz ellipsometer at the Terahertz Materials Analysis Center (THeMAC) at Linköping University was used.^[^ ^84^ ^]^ The permittivity extraction was realized by WVASE software (J. A. Woollam Co.), and the model used for conducting polymers was the Drude–Lorentz model .^[^ ^40^ ^]^

## Conflict of Interest

The authors declare no conflict of interest.

## Supporting information

Supporting Information

Supplemental Video 1

## Data Availability

The data that support the findings of this study are available from the corresponding author upon reasonable request.
