# Supplementary material for: Switchable Broadband Terahertz Absorbers Based on Conducting Polymer‐Cellulose Aerogels
Source: Adv Sci (Weinh). 2023 Nov 23;11(3):2305898. doi: 10.1002/advs.202305898 (PMC10797431; doi:10.1002/advs.202305898)
Supplement: Supplementary file 1 — Supporting Information [file ADVS-11-2305898-s001.pdf]

ADVANCED SCIENCE
Open Access

Supporting Information

for *Adv. Sci.*, DOI 10.1002/advs.202305898

Switchable Broadband Terahertz Absorbers Based on Conducting Polymer-Cellulose Aerogels

Chaoyang Kuang, Shangzhi Chen, Min Luo, Qilun Zhang, Xiao Sun, Shaobo Han, Qingqing Wang, Vallery Stanishev, Vanya Darakchieva, Reverant Crispin, Mats Fahlman, Dan Zhao, Qiye Wen* and Magnus P. Jonsson**

Supplementary information for

Switchable Broadband Terahertz Absorbers based on Conducting Polymer-Cellulose Aerogels

Chaoyang Kuang[1,†], Shangzhi Chen[1,†,*], Min Luo[2,†], Qilun Zhang[1,7], Xiao Sun[2], Shaobo Han[3], Qingqing Wang[1], Vallery Stanishev[4,5], Vanya Darakchieva[4,5], Reverant Crispin[1,3], Mats Fahlman[1,7], Dan Zhao[1], Qiye Wen[2,6,*], and Magnus P. Jonsson[1,7,8,*]

[1] Laboratory of Organic Electronics, Department of Science and Technology (ITN), Linköping University, Norrköping SE-601 74, Sweden
[2] School of Electronic Science and Engineering, State Key Laboratory of Electronic Thin Film and Integrated Devices, University of Electronic Science and Technology of China, Chengdu, Sichuan, 610 054, P. R. China
[3] School of Textile Material and Engineering, Wuyi University, 22 Dongchengcun, Jiangmen, Guangdong, 529 020, P. R. China
[4] Terahertz Materials Analysis Center (THeMAC) and Center for III-N Technology, C3NiT-Janzèn, Department of Physics, Chemistry and Biology (IFM), Linköping University, Linköping SE-581 83, Sweden
[5] Solid State Physics and NanoLund, Lund University, Lund SE-221 00, Sweden
[6] Yangtze Delta Region Institute (Huzhou), University of Electronic Science and Technology of China, Huzhou, Zhejiang, 313 001, P. R. China
[7] Wallenberg Wood Science Center, Linköping University, Norrköping SE-601 74, Sweden
[8] Stellenbosch Institute for Advanced Study (STIAS), Wallenberg Research Center at Stellenbosch University, Stellenbosch 7600, South Africa

[†] These authors contributed equally: Chaoyang Kuang, Shangzhi Chen, and Min Luo.
* Correspondence emails: magnus.jonsson@liu.se (Magnus P. Jonsson), qywen@uestc.edu.cn (Qi-Ye Wen), and shangzhi.chen@liu.se (Shangzhi Chen).

Supplementary Note I

XPS spectra of the aerogels at different redox states

XPS probes the element electronic structures of the aerogels' top surfaces, providing information on PEDOT:PSS doping level via the S2p core-level signal. The S2p spectrum of PEDOT:PSS consists of two doublets that can be attributed to PEDOT (at low binding energy of 167-163 eV) and PSS (at high binding energy of 171-167 eV), respectively (**Figure S16a**). For the aerogel in the pristine (or oxidized) state, the doublets of S2p core level signals are asymmetric due to the presence of positive charges delocalized over PEDOT chains[1]. Deconvolution of the PSS doublets indicates the existence of two types of sulfur atoms[2] for the pristine aerogel (top panel of **Figure S16a**). The higher binding energy parts (shaded by blue and brown) originate from the sulfonic acid groups of PSS^- (H/Na), while the lower binding energy parts (shaded by green and yellow) can be attributed to sulfonic groups of PSS^- interacting with $PEDOT^+$. After PEI vapour treatment (middle panel of **Figure S16a**), the peak positions for the PEDOT doublets shift to lower binding energies and their asymmetric feature is significantly suppressed (marked by purple arrow), indicating the reduction of PEDOT segments to their neutral states. This can result from the electron donation from residual monomer or small molecular impurities of PEI (*e.g.*, diethylenetriamine) and stabilized by the PSS^- forming equivalent salt complexes (**Figure S16b**). The conversion of PSS^- (H/Na) into PSS^-PEI^+ confirms the above hypothesis, as indicated by the shift of PSS^- (H/Na) doublets towards lower binding energies (marked by green dashed lines and black arrow in **Figure S16a**) yielding a PEDOT vs PSS S2p peak-to-peak binding energy difference in excellent agreement with previous studies[1]. As shown in the bottom panel of **Figure S16a**, the peak positions of PEDOT doublets shift back to higher binding energies and present an asymmetric feature after HCl vapour treatment, identical to its initial pristine oxidized state. In this scenario, HCl became the dominant acceptor of electrons (**Figure S16c**) due to its strong electrophilicity, leading to the re-oxidation of the neutral PEDOT segments.

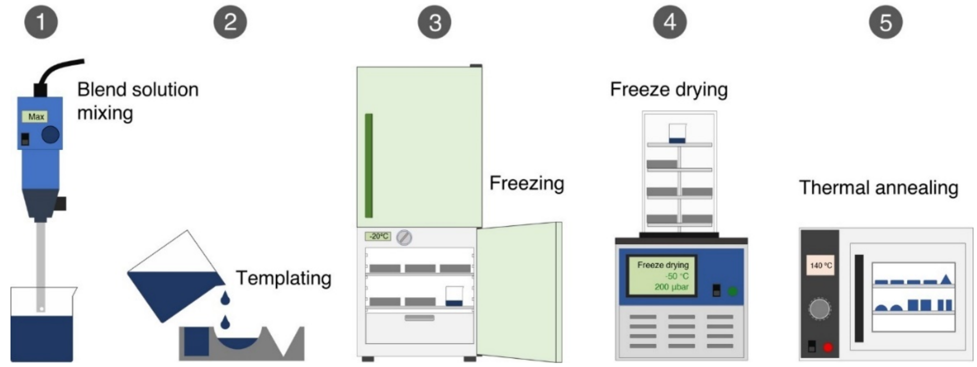

Figure S1 | The fabrication process flow of conducting polymer aerogels.

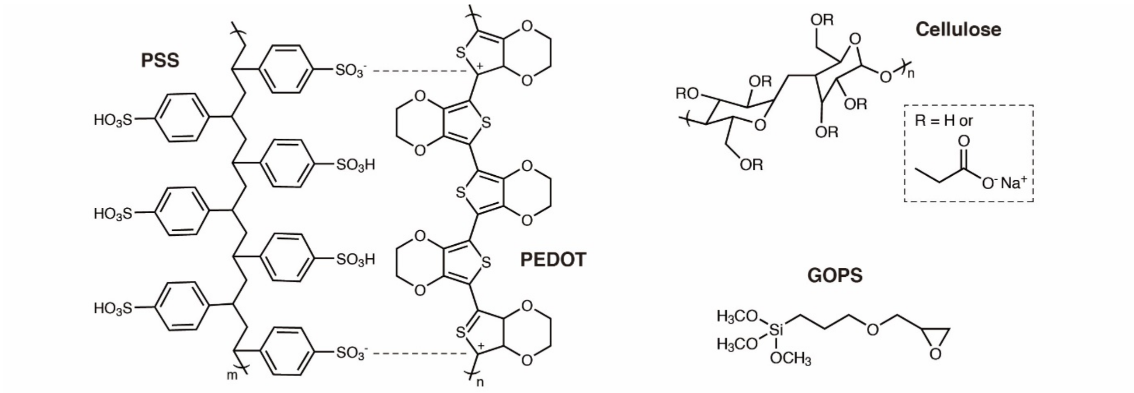

Figure S2 | Chemical structures of main components in conducting polymer aerogels. Cellulose provides the entire system with mechanical integrity. Without cellulose, pure PEDOT: PSS aerogels exhibit poor mechanical stability, where ashes or small particles keep falling off from the aerogels due to the relatively weak adhesion between PEDOT:PSS microstructures. (3-Glycidyloxypropyl)trimethoxysilane (GOPS) can crosslink PEDOT:PSS and cellulose[3] and thus effectively enhance the adhesion between the two components and the overall integrity. The concentration of GOPS in the precursor solution can affect the elasticity of the aerogels. Briefly, high-GOPS-concentration aerogels can rapidly recover to its original shape upon the release of external pressure/force[4].

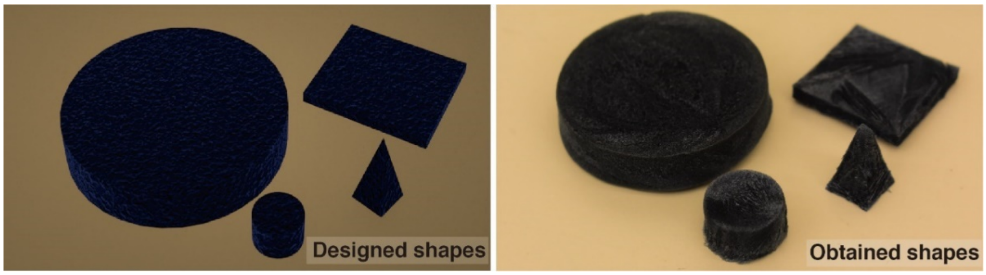

Figure S3 | Conducting polymer aerogels in various shapes made by different moulds.

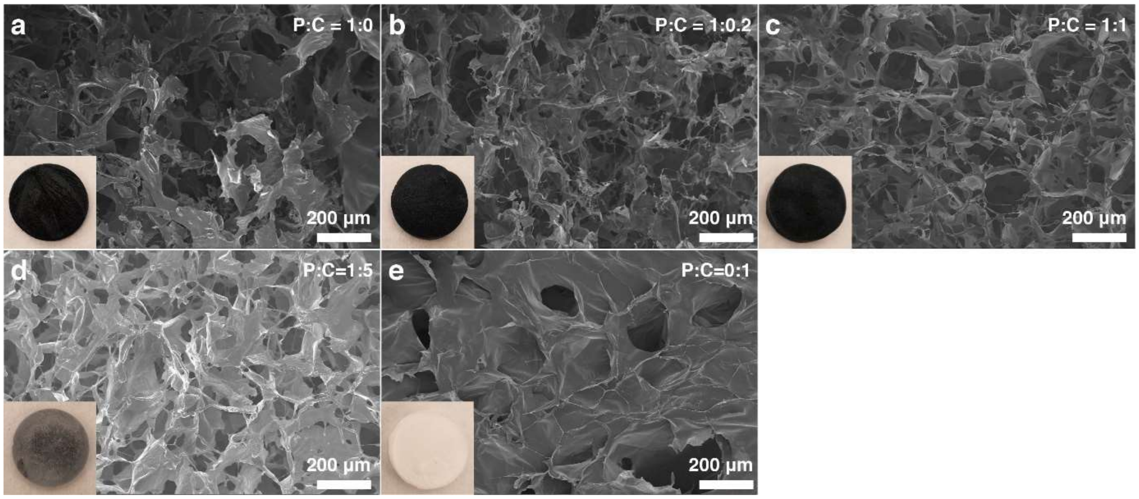

Figure S4 | Microstructural morphologies of conducting polymer aerogels with different ratios of PEDOT:PSS and cellulose. The ratio of PEDOT:PSS to cellulose varies from 1:0 to 0:1 as shown in the figure from **a** to **e**. Insets exhibit the sample images of the aerogels with corresponding ratios, where a clear trend of colour variation is presented. Scale bar for SEM images: 200 μm.

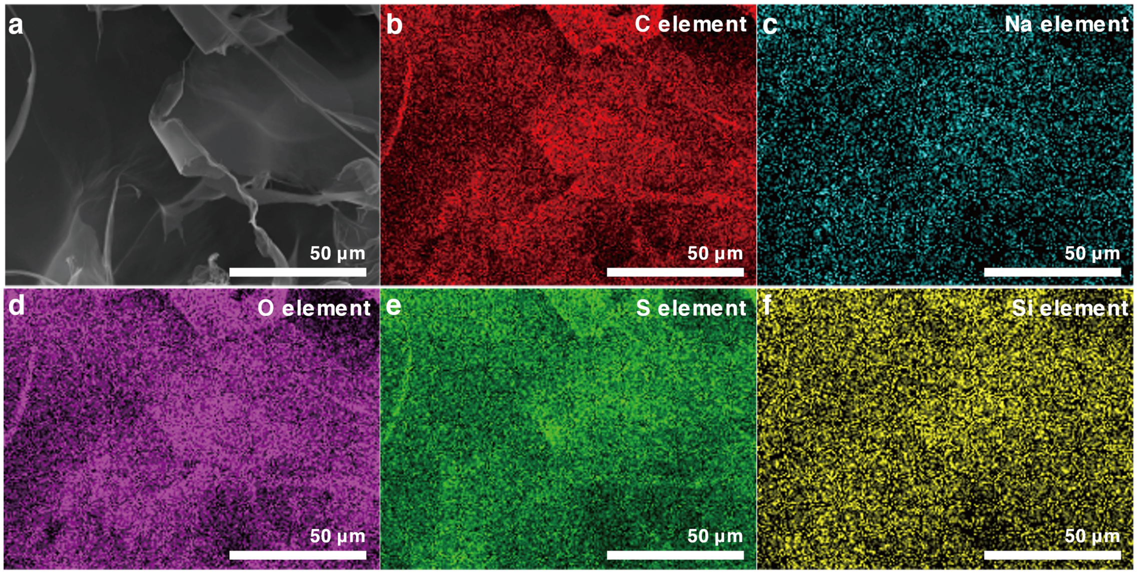

Figure S5 | EDS spectra of conducting polymer aerogels. **a**, SEM image of the probing area. **b-f**, Distribution of C (**b**), Na (**c**), O (**d**), S (**e**), and Si (**f**) elements. As shown in **Figure S2**, S, Na, and Si are the characteristic elements of PEDOT:PSS, cellulose, and GOPS. No phase segregation can be found, indicating the high homogeneity of the three components.

The PEDOT:PSS, cellulose, and GOPS ratio of the aerogel here was 1:1:0.2. Scale bar: 50 μm.

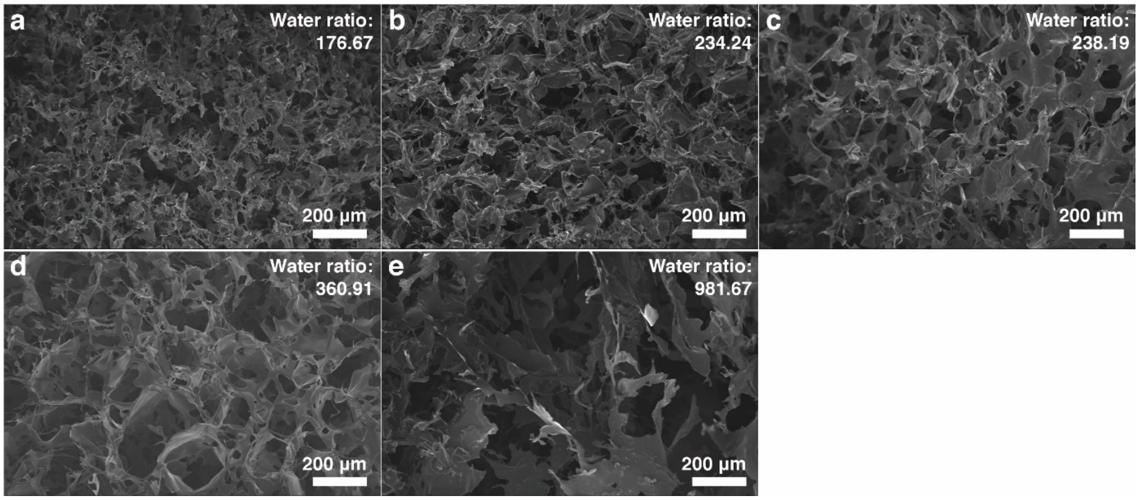

Figure S6 | Microstructural morphologies of conducting polymer aerogels with different water ratios. The water ratio is calculated by the weight ratio between water and PEDOT:PSS. A trend of increasing pore size can be observed with the increase of water ratio in precursor solution. For comparison, the ratio of PEDOT:PSS, cellulose, and GOPS was fixed as 1:1:0.2. Scale bar: 200 μm.

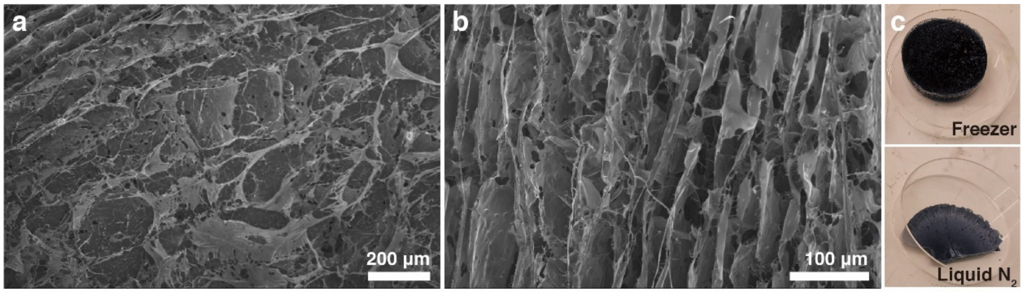

Figure S7 | Conducting polymer aerogels made by liquid nitrogen freezing method. **a** and **b**, Microstructural morphologies of the aerogels. **c**, Comparison between aerogels by two different freezing methods (freezer and liquid nitrogen). The ratio of PEDOT:PSS, cellulose, and GOPS was 1:1:0.2. Liquid nitrogen can freeze the precursor solution in a far shorter time (a few minutes) compared to the freezer method (4-5 hours). Thus, with liquid nitrogen freezing, water in precursor solution has limited time to form large ice crystals,

producing smaller pores in the samples. This can also be observed from the sample photos in **c**.

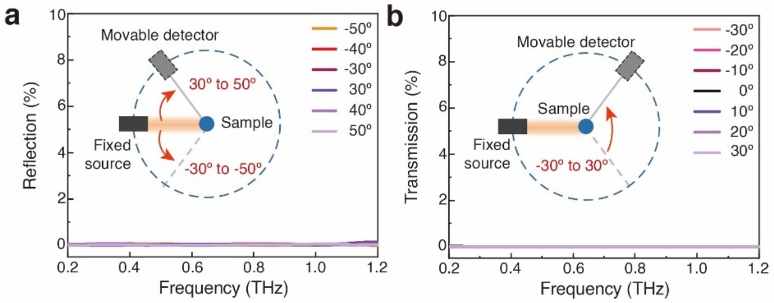

Figure S8 | Diffused reflection and transmission curves of conducting polymer aerogels. **a**, Diffused reflection with 6 different angles (-50°, -40°, -30°, 30°, 40°, and 50°). Inset: setup for the measurement. **b**, Diffused transmission with 7 different angles (-30°, -20°, -10°, 0°, 10°, 20°, 30°). Inset: setup for the measurement. In both measurements, the THz source was fixed and a movable THz detector was used. The used sample had a thickness of 6 mm and PEDOT:PSS, cellulose, GOPS ratio of 1:1:0.2. The effective detection area of the detector was about 506 mm^2 and the effective distance between sample and detector was about 100 mm. The average reflection for different angles range from 0.01 % to 0.04 %, while the average transmission range for all angles were below 0.002 %. Assuming the scattering is homogeneous in the hemisphere for the measurement, we can estimate the overall scattering for reflection and transmission to be below 4.96 % and 0.25 %. Thus, the scattered light only occupied a relatively small portion and most THz light was absorbed by the aerogel.

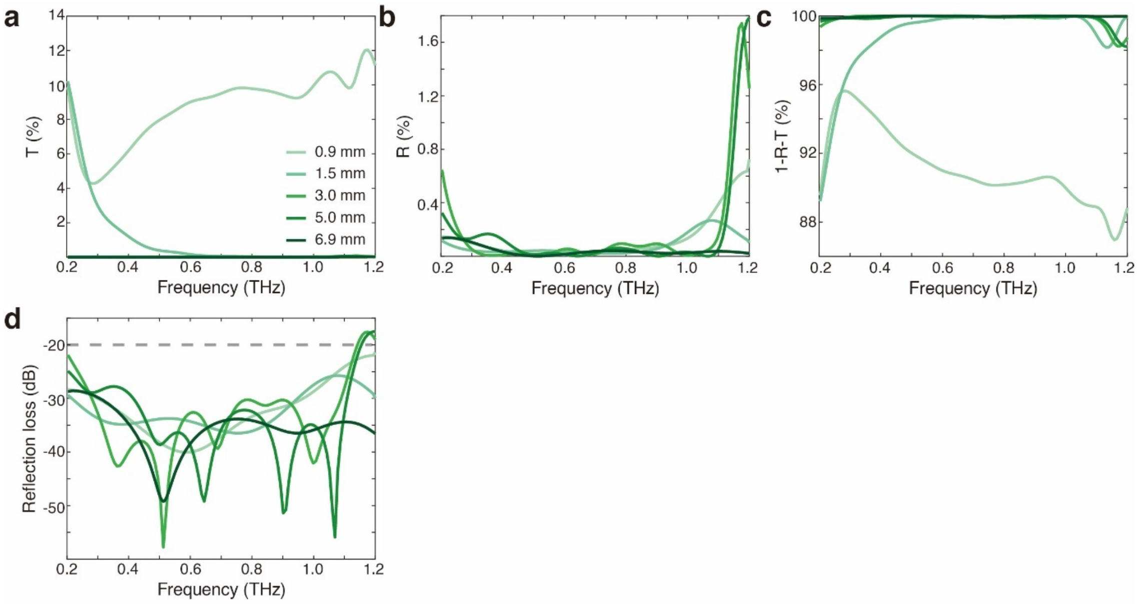

Figure S9 | THz properties of conducting polymer aerogels with different thicknesses. **a**, Transmission (T) curves. **b**, Specular reflection (R) curves. **c**, Absorption (1-R-T) curves. **d**, Reflection loss curves. The aerogels used here had a PEDOT:Cellulose:GOPS ratio of 1:1:0.2. The thicknesses from light colours to dark colours were 0.9 mm, 1.5 mm, 3.0 mm, 5.0 mm, and 6.9 mm, respectively.

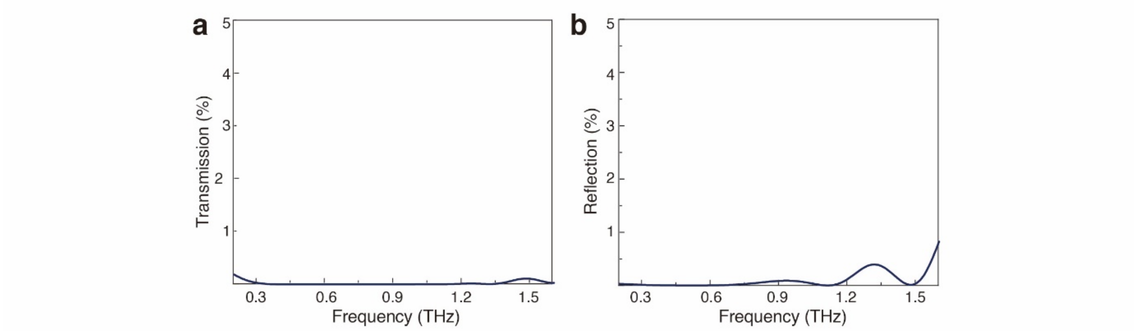

Figure S10 | Transmission (a) and specular reflection (b) of conducting polymer aerogels in the range between 0.2 and 1.6 THz. The aerogel presented here is the one used in **Figure 2** but presented with broad ranges. The aerogels show increased reflection and transmission above 1.2 THz, leading to the absorption (1-R-T) less than 99 % (with RL > -20 dB). Thus, the qualified absorption bandwidth for the aerogels is 1.4 THz (from 0.2 to 1.6 THz), covering the whole measurement range.

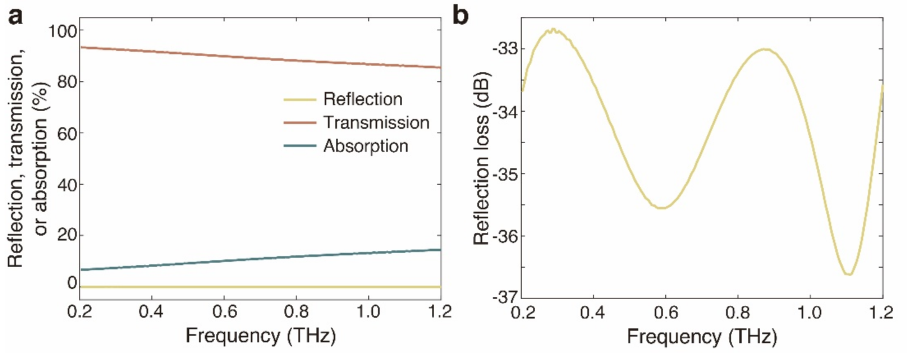

Figure S11 | THz properties of a pure cellulose aerogel. **a**, Transmission (T), specular reflection (R), and absorption (1-R-T) curves of the pure cellulose aerogels. **b**, Reflection loss curves. The thickness for the sample was 6 mm.

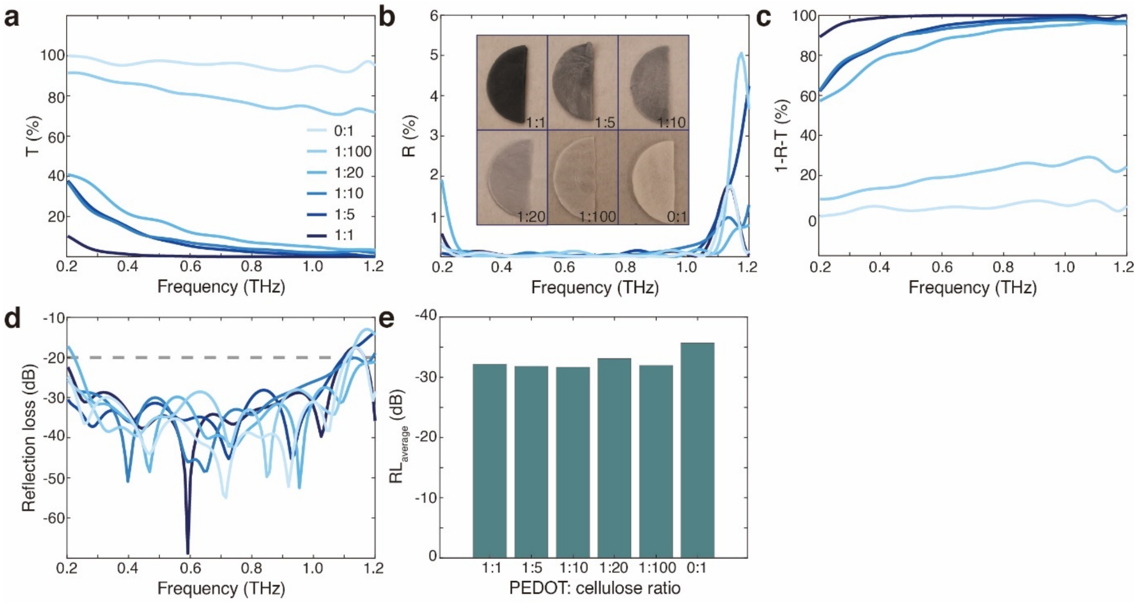

Figure S12 | THz properties of conducting polymer aerogels in different material ratios. **a**, Transmission (T) curves. **b**, Specular reflection (R) curves. **c**, Absorption (1-R-T) curves. **d**, Reflection loss curves. **e**, $RL_{average}$ comparison. Images of the aerogel samples are shown in the inset of **b**. The thickness of all aerogels is 1.5 mm. The ratio between PEDOT:PSS and GOPS was 1:0.2. The ratios between PEDOT:PSS and cellulose were 1:1, 1:5, 1:10, 1:20, and 1:100.

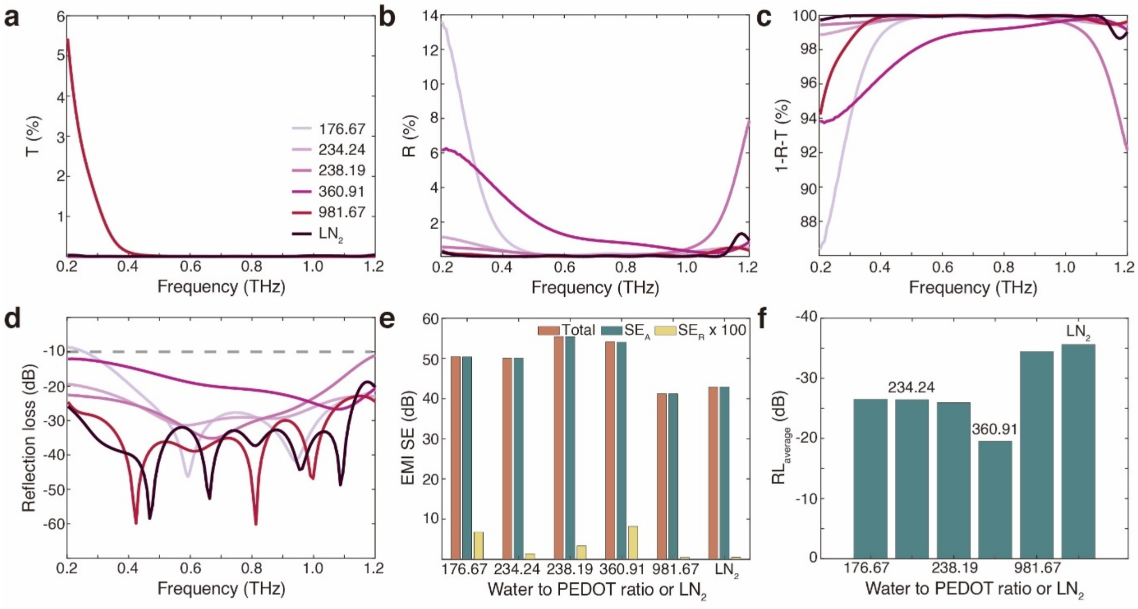

Figure S13 | THz properties of conducting polymer aerogels with different pore sizes (*via* different water concentrations or liquid nitrogen cooling). **a**, Transmission (T) curves. **b**, Specular reflection (R) curves. **c**, Absorption (1-R-T) curves. **d**, Reflection loss curves. **e**, EMI SE comparison. **f**, $RL_{average}$ comparison. The thickness of all aerogels was 6 mm. The ratio of PEDOT, cellulose, and GOPS was 1:1:0.2. Ratios of water to PEDOT:PSS were 176.67, 234.24, 238.19, 360.91, and 981.67. For liquid nitrogen cooled sample (LN_2), ratio of water to PEDOT:PSS was 174.86.

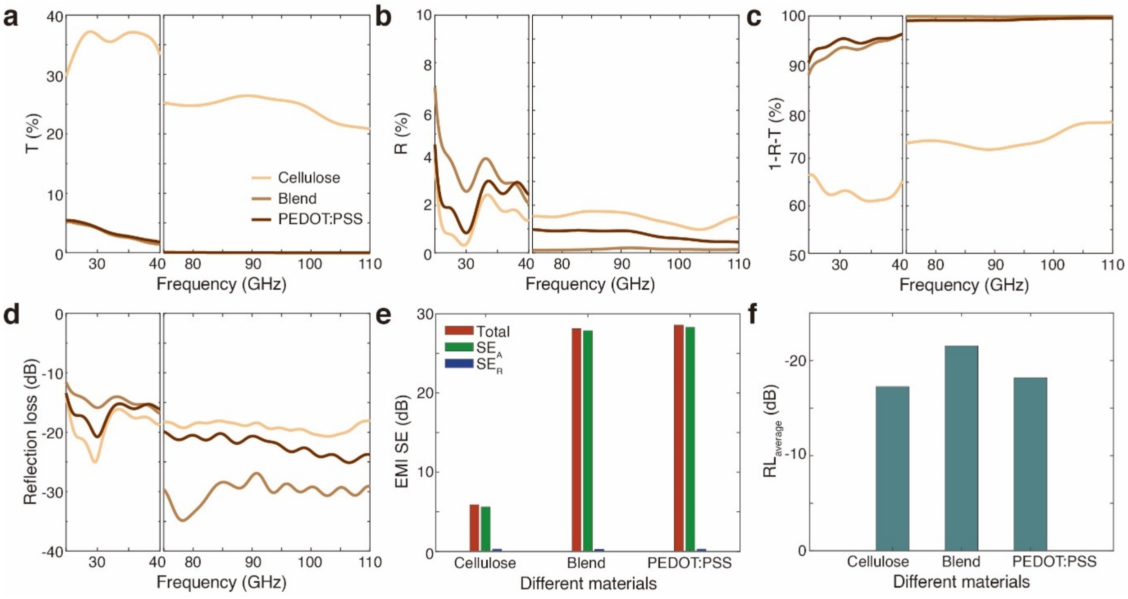

Figure S14 | Microwave properties of conducting polymer aerogels made of different materials. a, Transmission (T) curves. **b**, Specular reflection (R) curves. **c**, Absorption (1-R-T) curves. **d**, Reflection loss curves. **e**, $RL_{average}$ comparison. The thickness of all aerogels was 6 mm. The ratio of PEDOT, cellulose, and GOPS for blend was 1:1:0.2.

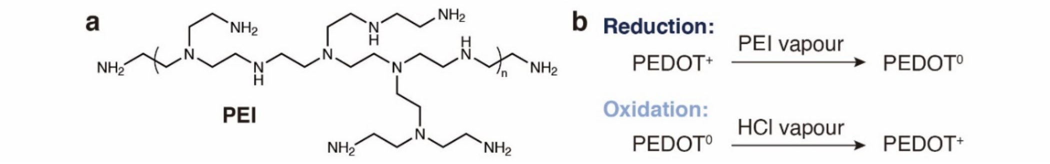

Figure S15 | Redox tuning mechanism of conducting polymers. a, Chemical structure of branched PEI. **b**, The redox state (doping) modulation with PEI and HCl vapour treatments, where PEDOT can be transitioned between electrical insulators and conductors.

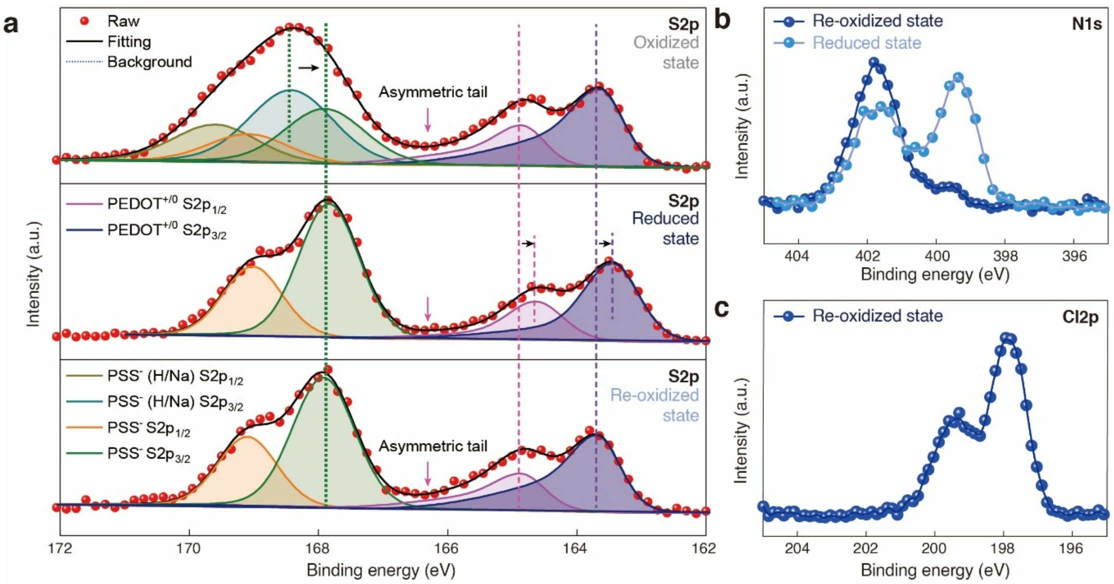

Figure S16 | XPS spectra of the conducting polymer aerogels at different redox states. **a**, S2p spectra. **b**, N1s spectra. **c**, Cl2p spectra. The aerogel used here has a thickness of 1.5 mm and PEDOT:PSS to cellulose ratio of 1:10.

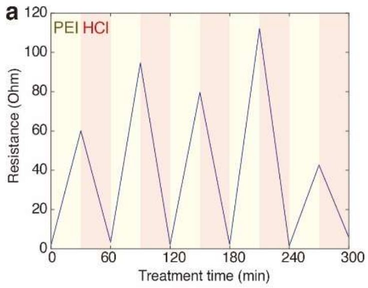

Figure S17 | Cycling electrical resistance characterizations of the conducting polymer aerogels with alternating PEI and HCl vapour treatments. **a**, Single treatment time of 30 min. **b**, Single treatment time of 10 min. The aerogel used here had a thickness of 1.5 mm and PEDOT:PSS to cellulose ratio of 1:10.

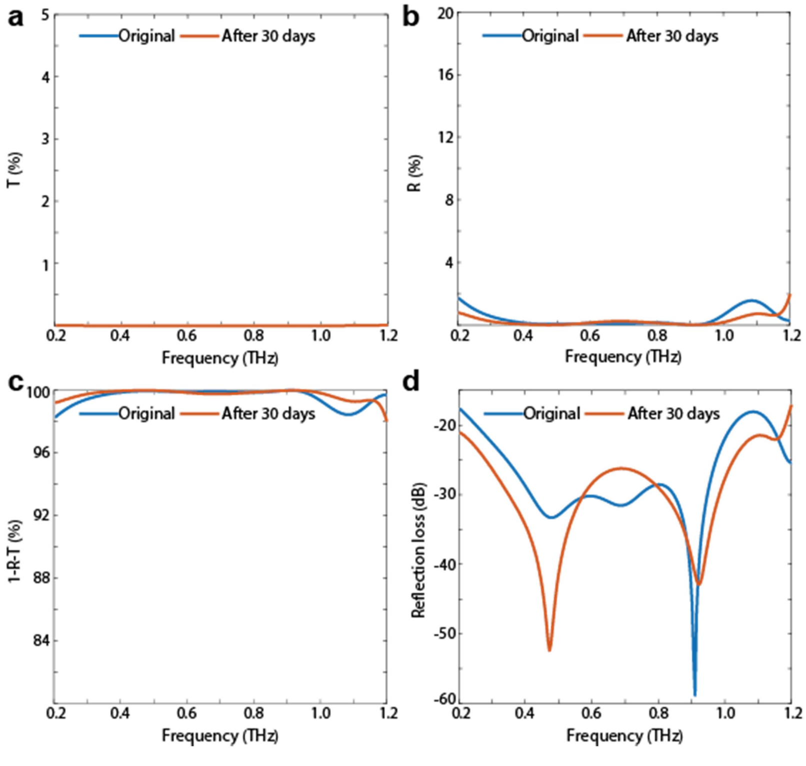

Figure S18 | Optical memory effect of an oxidized conducting polymer aerogel. **a**, Transmission (T). **b**, Reflection (R). **c**, Absorption (1-R-T). **d**, Reflection loss. The sample used here possessed the ratio of PEDOT:PSS to cellulose of 1:1 and the thickness of 6 mm. It was kept in atmosphere for 30 days without showing drastic change in optical properties.

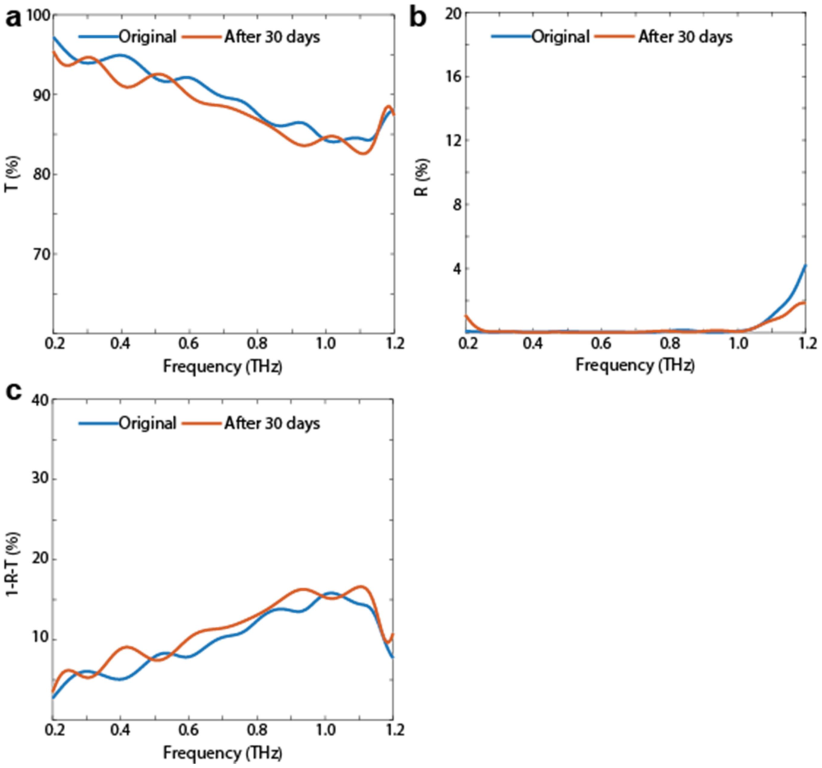

Figure S19 | Optical memory of a PEI-vapour reduced conducting polymer aerogel. a, Transmission (T). **b**, Reflection (R). **c**, Absorption (1-R-T). The reduced samples were kept in atmosphere for 30 days presenting similar optical behaviours, indicating its excellent stability and memory effect. The ratio of PEDOT:PSS and cellulose was 1:10, and the

thickness was 1.5 nm (the THz behavior of this sample in oxidized state was shown in **Figure 3d** and **Figure S20b**).

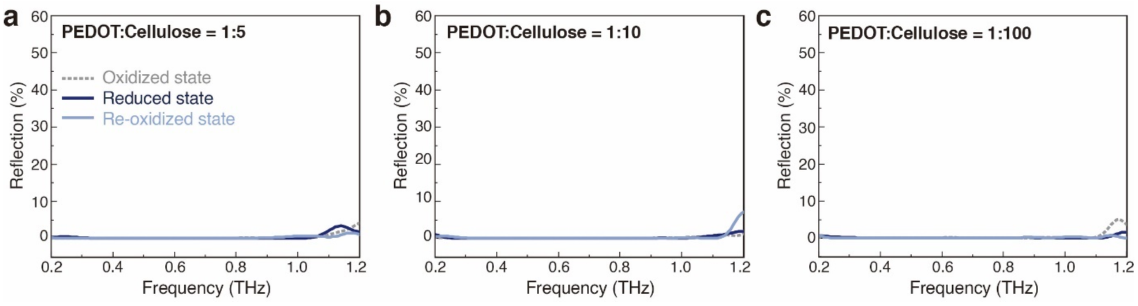

Figure S20 | THz specular reflection spectra of conducting polymer aerogels at different redox states. The ratio of PEDOT:PSS and cellulose is varied from 1:5 (**a**), 1:10 (**b**), to 1:100 (**c**). All samples present a negligible specular reflection due to the porous microstructures of the aerogels. The thickness of all aerogels used above was 1.5 mm.

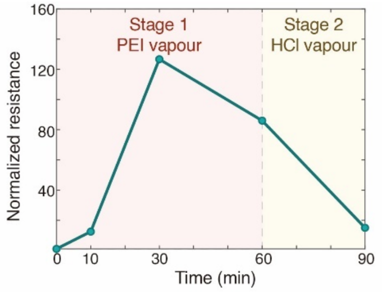

Figure S21 | Normalized electrical resistance variation of aerogels with PEI and HCl vapour treatment. The aerogel has a PEDOT:PSS to cellulose ratio of 1:100.

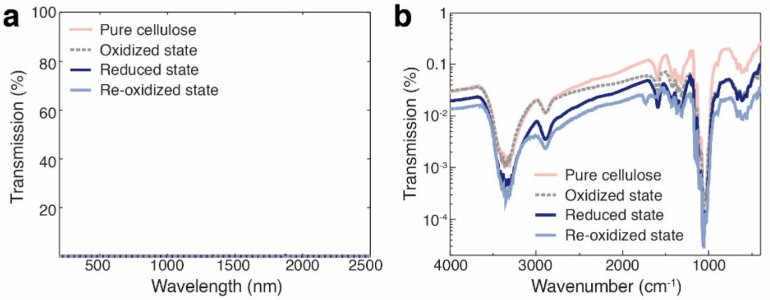

Figure S22 | Visible-NIR (a) and MIR (b) transmission of conducting polymer aerogels at different redox states. For comparison, pure cellulose aerogel is used as reference. The optical transmission (with normal incidence) did not show obvious changes at different redox states in these ranges. The aerogel used here had a thickness of 1.5 mm and PEDOT:PSS to cellulose ratio of 1:10.

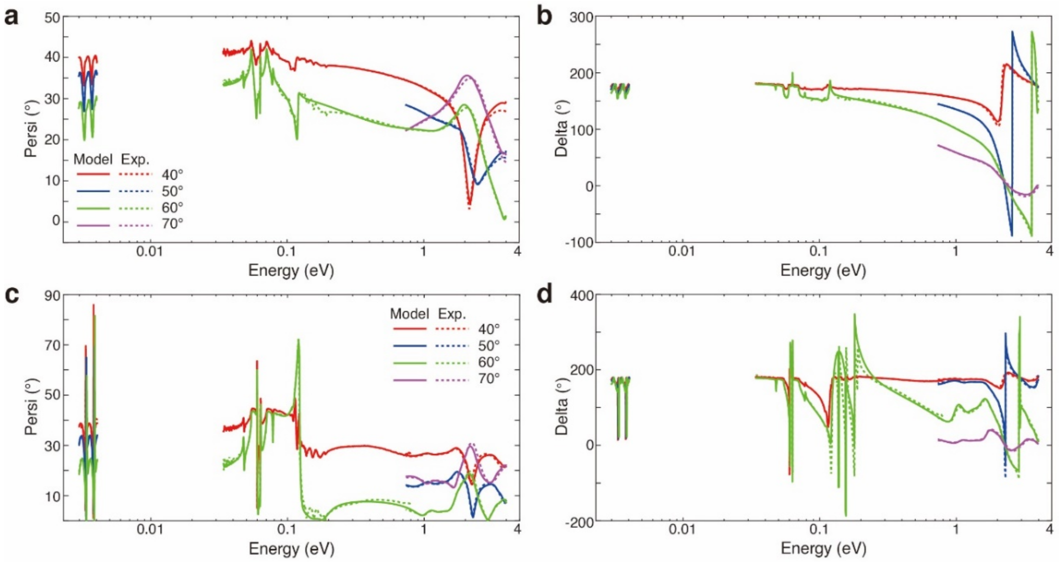

Figure S23 | Ellipsometric raw data for PEDOT:PSS films. **a** and **b** are for PEDOT:PSS-GOPS films without PEI vapour treatment, while **c** and **d** are for PEDOT:PSS-GOPS films with PEI vapour treatment. The experimental data (dashed lines) are fitted with the Drude-Lorentz model used in our previous studies[5]. Briefly, the Drude component in the model is responsible for the electrical charge carrier transport, while the Lorentz oscillators are included to describe other physical processes, such as interband transitions and molecular vibrations.

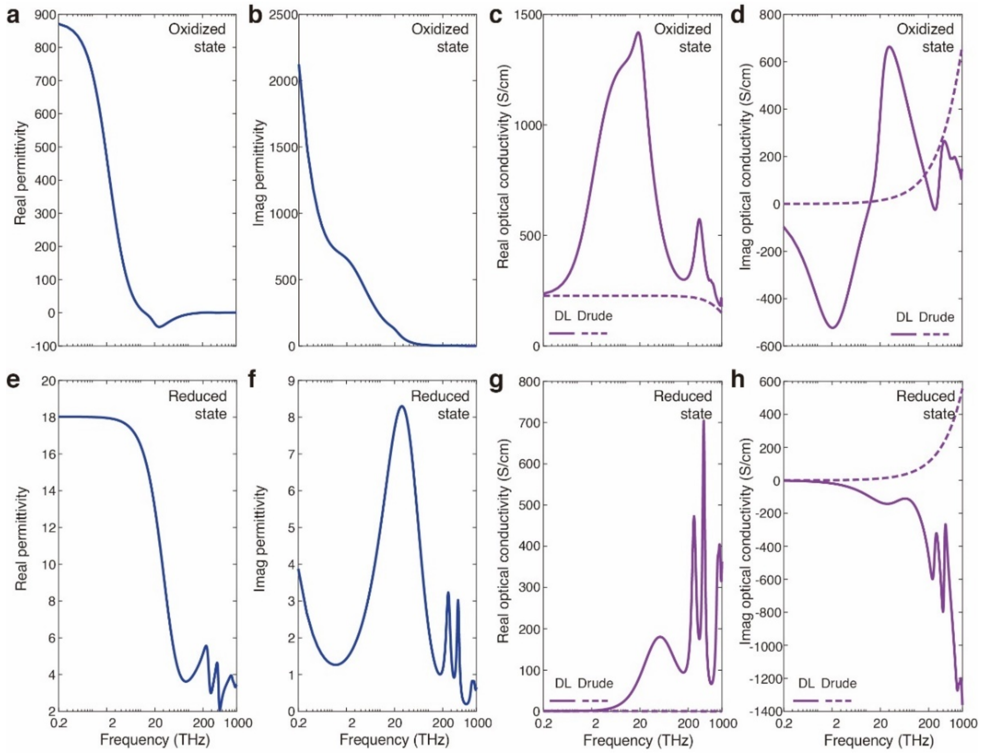

Figure S24 | Permittivity and optical conductivity dispersions of PEDOT:PSS-GOPS films with and without PEI vapour treatment. The top panel is for PEDOT:PSS-GOPS film and the bottom panel is for PEI-treated PEDOT:PSS-GOPS film. For permittivity and optical conductivity, both the real and imaginary parts are presented. The Drude component of the Drude-Lorentz model is presented in dashed lines for optical conductivity dispersion.

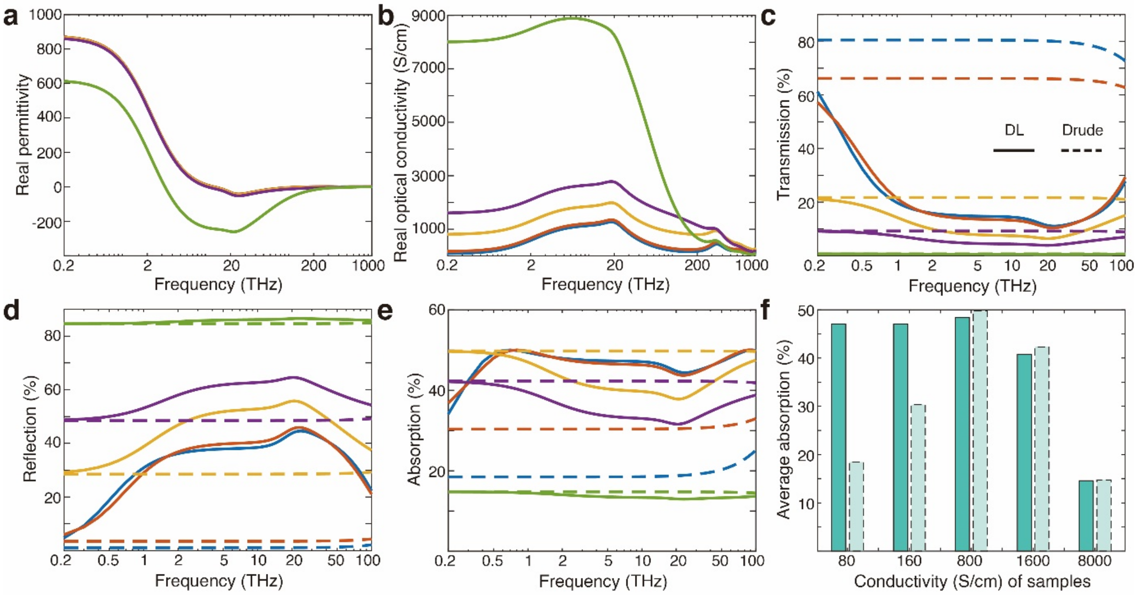

Figure S25 | THz and far infrared properties of artificial materials with different optical conductivity dispersion. The real permittivity (**a**), real optical conductivity (**b**), and calculated transmission (**c**), reflection (**d**), absorption (**e**), and average absorption in THz range (**f**) of five artificial materials are presented. The optical parameters of the five materials were generated using the Drude-Lorentz (DL) and the Drude model, possessing DC conductivities (angular frequency of 0) of 80 (blue), 160 (orange), 800 (yellow), 1600 (purple), and 8000 (green) S/cm, respectively. The changes of DC conductivities here are made by varying the charge carrier mobility μ (varying the charge carrier density n can achieve similar results). For each panel, full lines or bars correspond to Drude-Lorentz model, while dashed lines or bars correspond to only having the Drude term. More details can be found in our previous study[6].

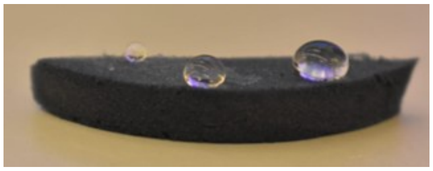

Figure S26 | The aerogel remains hydrophobic after 4 weeks. The aerogel had a thickness of 6 mm and PEDOT:PSS to cellulose ratio of 1:1.

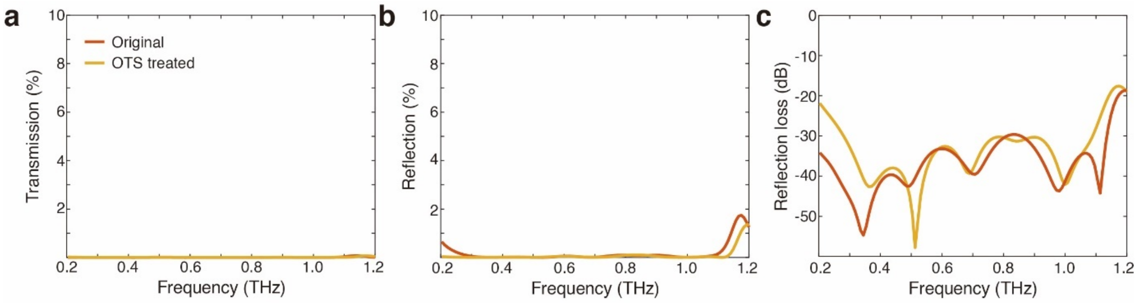

Figure S27 | Transmission, specular reflection, and RL of OTS vapour-treated aerogels.

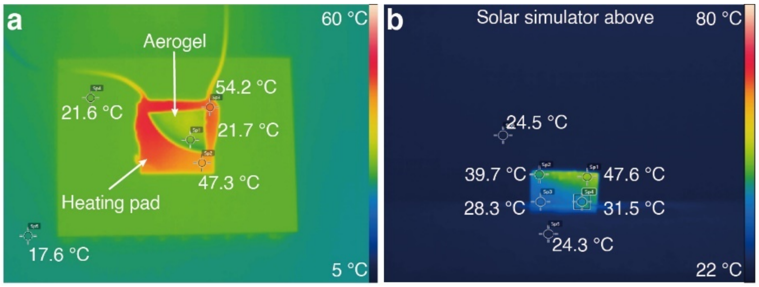

Figure S28 | Thermal camera images of aerogels. a, The aerogel placed on a heating pad. **b**, The aerogel illuminated by a solar simulator. The aerogel shows a low thermal conductivity and the illumination-induced heat is mostly localized in the top surface (T approaching 50 °C).

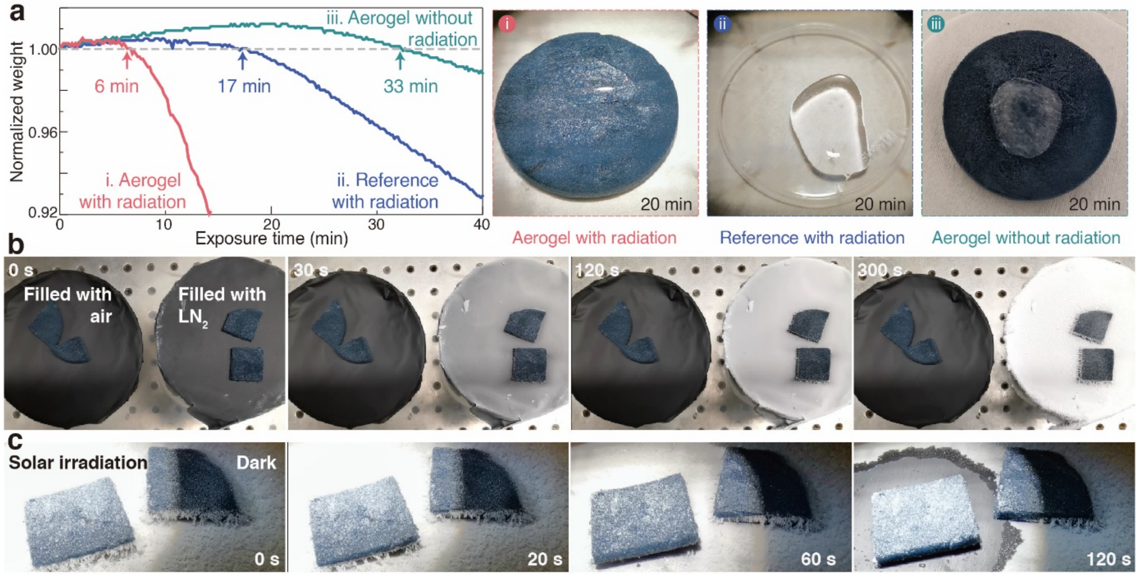

Figure S29 | Simulated de-icing, frosting, and de-frosting processes of conducting polymer aerogels. a, Normalized weight variation curves of aerogel and reference samples

with ice pieces (weight of about 1 g) on top under different situations (i, aerogel with radiation; ii, reference with radiation; and iii, aerogel without radiation). Images of the samples exposed to solar radiation of 20 minutes are exhibited in the right panel. **b**, The frosting process for the aerogels. The aerogels are placed on top of a beaker filled with liquid nitrogen (right one). Signs of frost direct emerge at about 5 seconds and it takes in total 300 seconds to form a thick frost layer. **c**, De-frosting process of the aerogels within 2 minutes.

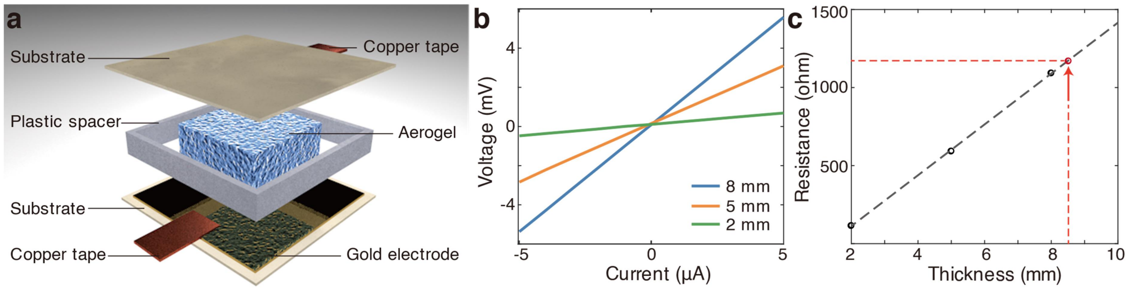

Figure S30 | Electrical resistance measurement of the aerogels. a, The measurement set-up. **b**, The measured I-V curves of the aerogel with different spacer thickness. **c**, Interpolation of the electrical resistance of the aerogel at its original thickness. The used aerogel here has a thickness of 8.48 mm. The interpolated electrical resistance is about 1200 ohm.

Table S1 | The composition ratio, electrical, and THz shielding properties of conducting polymer aerogels. The weight ratios of cellulose, GOPS, and deionized water are normalized based on the weight of PEDOT:PSS. t*D is the product of aerogel density and thickness.

No.	Weight ratio				Cooling method	Conductivity (mS/cm)	t*D (mg/cm^2)	Max SSE/t (dB cm^2/g)
	PEDOT	Cellulose	GOPS	Water				
1	1	0.00	0.00	75.92	Freezer	0.1027	11.13	5374.46
2	1	0.97	0.00	210.55	Freezer	0.1556	5.28	13225.56
3	1	0.98	0.23	183.09	Freezer	0.2399	9.06	6037.29
4	1	0.97	0.19	277.11	Freezer	0.0884	5.46	11261.70
5	1	1.00	0.27	238.19	Freezer	0.0864	6.67	10433.63
6	1	1.00	0.20	360.91	Freezer	0.0609	4.82	15360.81
7	1	1.00	0.20	589.52	Freezer	0.0125	1.80	26441.39
8	1	5.02	0.22	490.00	Freezer	0.0067	1.05	66493.99
9	1	5.00	0.22	1112.40	Freezer	0.0052	0.98	64508.47
10	1	10.02	0.23	2148.90	Freezer	0.0023	0.67	34838.15
11	1	0.97	0.25	297.84	Liquid N_2	0.0437	6.19	9427.46
12	1	2.09	0.22	194.72	Liquid N_2	0.1343	13.17	3963.79

Table S2 | Properties comparison between current state-of-the-art THz absorbers. Our conducting polymer aerogels are compared with graphene/PMMA nanolaminates[7], graphene foams[8], MXene forms[9] and graphene devices[10] reported in literature, based on six attributes. For cost comparison, the prices are collected from Sigma-Aldrich (www.sigmaaldrich.com).

Attributes	Conducting polymer aerogels	Graphene/PMMA nanolaminates[7]	Graphene foams[8]	MXene foams[9]	Graphene devices[10]
Qualified Bandwidth (measured bandwidth)	1.4 THz (1.4 THz)	1.8 THz (1.8 THz)	1.5 THz (1.5 THz)	1.1 THz (1.8 THz)	Not indicated
SSE/t	6.6×10^4 $dB{\cdot}cm^2{\cdot}g^{-1}$	3.0×10^5 $dB{\cdot}cm^2{\cdot}g^{-1}$	1.1×10^5 $dB{\cdot}cm^2{\cdot}g^{-1}$	5.5×10^4 $dB{\cdot}cm^2{\cdot}g^{-1}$	Not indicated (but the device has a metal backplate)
$RL_{average}$	-39.5 dB	~ -10 dB	~ -30 dB	~ -10 dB	Low (highly reflective surface)
Sustain-ability	Cellulose are natural materials[11], PEDOT:PSS is widely used for in-vivo applications[12,13]	Graphene has potential risk to respiratory systems[14] and cell mebranes[15]	Graphene has potential risk to respiratory systems[14] and cell mebranes[15], carbon nanotubes can be toxic[16]	Toxic, corrosive, non-eco-friendly	Graphene has potential risk to respiratory systems[14] and cell mebranes[15], lithium nickel manganese cobalt oxide might cause explosion[17]
Ease of fabrication	Solution mixing of PEDOT:PSS and cellulose, followed by freeze-drying and intermediate temperature annealing (140 °C)	Chemical vapour deposition for graphene and spin coating for PMMA, repeating above steps for 4-100 times	Modified solvothermal reaction for graphene flakes, freeze-drying, followed by high temperature annealing (400-1500 °C)	LiF and HCl etching of Ti_3AlC_2 powder to obtain MXene, modified solvothermal reaction for graphene flakes, HCl/Zn foil gelation (2 days), freeze-drying	Chemical vapour deposition for graphene on Ni foil substrate at 1050 °C; Ni foil etching by $FeCl_3$
Cost effective-ness	~260 Euro/250 g for PEDOT:PSS ~250 Euro/1 kg for cellulose	~500 Euro/1 kg for PMMA ~440 Euro/500 mg for graphene	~440 Euro/500 mg for graphene ~380 Euro/ 5 g multi-walled carbon tubes	~210 Euro/25 g for Ti_3AlC_2 powder ~440 Euro/500 mg for graphene	~440 Euro/500 mg for graphene; ~185 Euro/10 g for lithium nickel manganese cobalt oxide
Switch-ability	Average transmission: 13 % to 91 %	No switchability	No switchability	No switchability	Average reflection: 50 % to 85 %

Video S1 | Conducting polymer aerogels after OTS treatments. As shown in the video, the water droplets can easily be removed away from the aerogel surface due to the hydrophobic surface. Water cannot penetrate into the aerogel, making it float on the water bath with a dry surface. 2x speed was used in the video recording.

References

1 Fabiano, S. *et al.* Poly (ethylene imine) impurities induce n‐doping reaction in organic (semi) conductors. *Advanced Materials* **26**, 6000-6006 (2014).

2 Greczynski, G. *et al.* Photoelectron spectroscopy of thin films of PEDOT–PSS conjugated polymer blend: a mini-review and some new results. *Journal of Electron Spectroscopy and Related Phenomena* **121**, 1-17 (2001).

3 Håkansson, A. *et al.* Effect of (3‐glycidyloxypropyl) trimethoxysilane (GOPS) on the electrical properties of PEDOT: PSS films. *Journal of Polymer Science Part B: Polymer Physics* **55**, 814-820 (2017).

4 Han, S. *et al.* Cellulose‐conducting polymer aerogels for efficient solar steam generation. *Advanced Sustainable Systems* **4**, 2000004 (2020).

5 Chen, S. *et al.* On the anomalous optical conductivity dispersion of electrically conducting polymers: Ultra-wide spectral range ellipsometry combined with a Drude–Lorentz model. *Journal of Materials Chemistry C* **7**, 4350-4362 (2019).

6 Karki, A. *et al.* Electrical Tuning of Plasmonic Conducting Polymer Nanoantennas. *Advanced Materials* **34**, 2107172 (2022).

7 Pavlou, C. *et al.* Effective EMI shielding behaviour of thin graphene/PMMA nanolaminates in the THz range. *Nature Communications* **12**, 1-9 (2021).

8 Huang, Z. *et al.* Graphene‐Based Composites Combining Both Excellent Terahertz Shielding and Stealth Performance. *Advanced Optical Materials* **6**, 1801165 (2018).

9 Lin, Z. *et al.* Highly stable 3D Ti3C2T x MXene-based foam architectures toward high-performance terahertz radiation shielding. *ACS Nano* **14**, 2109-2117 (2020).

10 Ergoktas, M. S. *et al.* Multispectral graphene-based electro-optical surfaces with reversible tunability from visible to microwave wavelengths. *Nature Photonics* **15**, 493-498 (2021).

11 Zhao, D. *et al.* Cellulose‐based flexible functional materials for emerging intelligent electronics. *Advanced Materials* **33**, 2000619 (2021).

12 Khodagholy, D. *et al.* In vivo recordings of brain activity using organic transistors. *Nature Communications* **4**, 1-7 (2013).

13 Simon, D. T., Gabrielsson, E. O., Tybrandt, K. & Berggren, M. Organic bioelectronics: bridging the signaling gap between biology and technology. *Chemical Reviews* **116**, 13009-13041 (2016).

14 Schinwald, A., Murphy, F. A., Jones, A., MacNee, W. & Donaldson, K. Graphene-based nanoplatelets: a new risk to the respiratory system as a consequence of their unusual aerodynamic properties. *ACS Nano* **6**, 736-746 (2012).

15 Tu, Y. *et al.* Destructive extraction of phospholipids from Escherichia coli membranes by graphene nanosheets. *Nature Nanotechnology* **8**, 594-601 (2013).

16 Liu, Y., Zhao, Y., Sun, B. & Chen, C. Understanding the toxicity of carbon nanotubes. *Accounts of Chemical Research* **46**, 702-713 (2013).

17 Barone, T. L. *et al.* Lithium-ion battery explosion aerosols: Morphology and elemental composition. *Aerosol Science and Technology* **55**, 1183-1201 (2021).
